# Promising Sources of Plant-Derived Polyunsaturated Fatty Acids: A Narrative Review

**DOI:** 10.3390/ijerph20031683

**Published:** 2023-01-17

**Authors:** Gianluca Rizzo, Luciana Baroni, Mauro Lombardo

**Affiliations:** 1 Independent Researcher, Via Venezuela 66, 98121 Messina, Italy; 2 Scientific Society for Vegetarian Nutrition, 30171 Venice, Italy; 3 Department of Human Sciences and Promotion of the Quality of Life, San Raffaele Open University, 00166 Rome, Italy

**Keywords:** fatty acids, omega-3, alpha-linolenic acid, docosahexaenoic acids, eicosapentaenoic acid, stearidonic acid, microalgae, seaweed, fish oils, diet, vegetarian, unsaturated

## Abstract

(1) Background: Polyunsaturated fatty acids (PUFAs) are known for their ability to protect against numerous metabolic disorders. The consumption of oily fish is the main source of PUFAs in human nutrition and is commonly used for supplement production. However, seafood is an overexploited source that cannot be guaranteed to cover the global demands. Furthermore, it is not consumed by everyone for ecological, economic, ethical, geographical and taste reasons. The growing demand for natural dietary sources of PUFAs suggests that current nutritional sources are insufficient to meet global needs, and less and less will be. Therefore, it is crucial to find sustainable sources that are acceptable to all, meeting the world population’s needs. (2) Scope: This review aims to evaluate the recent evidence about alternative plant sources of essential fatty acids, focusing on long-chain omega-3 (n-3) PUFAs. (3) Method: A structured search was performed on the PubMed search engine to select available human data from interventional studies using omega-3 fatty acids of non-animal origin. (4) Results: Several promising sources have emerged from the literature, such as algae, microorganisms, plants rich in stearidonic acid and GM plants. However, the costs, acceptance and adequate formulation deserve further investigation.

## 1. Introduction

### 1.1. Chemical Structures of Essential Fatty Acids and Dietary Sources

Essential fatty acids (EFAs) belong to a class of indispensable lipid macromolecules. Humans are unable to synthesize them de novo and they need to introduce EFAs via the diet. These molecules contain multiple double bonds in their carbonaceous scaffold, and the position of the first double bond on the aliphatic chain, proximal to the terminal methyl group (omega), defines the two subclasses of EFAs: omega 3 polyunsaturated fatty acids (n3 PUFAs) and omega 6 polyunsaturated fatty acids (n6 PUFAs) [1].

In foodstuff, essential fatty acids can be found in the form of both long-chain (with 20 or more carbons) and in the form of precursors or short-chain, such as alpha-linolenic acid (C18:3 cis-9,12,15; ALA) belonging to subclass n3 PUFAs, and linoleic acid (C18:2 cis-9,12; LA) belonging to the subclass of n6 PUFAs [2,3]. Precursors, especially molecules belonging to the n6 PUFAs subclass, are found in significant quantities in plant foods, including fruits, seeds and oils. Flaxseeds are particularly rich in ALA with 39–64% of total fat and with a lipid fraction up to 50% *w*/*w* [4,5]. Another example is soybean oil, which contains up to 54% of LA [6]. Table 1 shows the concentrations of EFAs in selected plant foods and derived oils.

**Figure 1:**
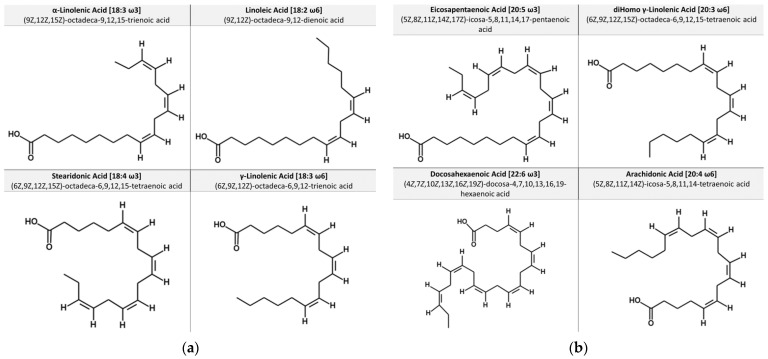
Chemical structures of selected essential fatty acids: (**a**) short-chain essential fatty acids; (**b**) long-chain essential fatty acids.

Humans are unable to use the aforementioned precursors and need to transform them into long-chain fatty acids (LC-PUFAs) using elongation and desaturation steps, which involve reactions catalyzed by elongase and desaturase enzymes. They can be obtained in pre-formed and ready-to-use forms through the diet. In this context, only ALA and LA can be strictly defined as essential [8]. The most representative molecules of the LC-PUFAs are eicosapentaenoic acid (20:5 cis-5,8,11,14,17; EPA) and docosahexaenoic (C22:6 cis-4,7,10,13,16,19; DHA), which belong to the subclass of n3 PUFAs, and arachidonic acid (C20:4 cis-5,8,11,14; AA), which belongs to the subclass of n6 PUFAs. Food sources of LC-PUFAs are mainly of animal origin, such as breast milk, and fatty fish such as salmon and small blue fish such as anchovies, sardines, mackerel, herring and eel. They may also be found, although to a lesser extent, in the meat of land animals [9,10]. Some plant foods, such as seaweed, can contain pre-formed EFAs in varying concentrations. On the other hand, terrestrial plants are unable to produce LC-PUFAs [11]. Figure 1 shows the molecular structures of the main essential fatty acids, including IUPAC names.

### 1.2. Functions and Biological Activities of Essential Fatty Acids

EFAs perform various biological functions. Their selective incorporation into cell membranes contributes to their fluidity and thus modulates intercellular communication. Some anatomical structures show a particular enrichment in EFAs in membrane phospholipids, such as in the central nervous system and retina, where LC-PUFAs modulate nerve transmission and the activation of rhodopsin for vision, respectively [12,13,14]. n3 PUFAs could be implicated in cognitive mechanisms both in the early stages of development and during the cognitive decline in senescence, with direct roles in the enrichment of the cell membranes of the nervous system and consequent functions [15], but also indirectly, through their metabolic role in the metabolism of B vitamins, which in turn are involved in cognition [16].

LC-PUFAs’ catabolism plays a crucial role in the molecular action of these compounds. Oxidation processes catalyzed by the enzymes lipoxygenases (LOX), cyclooxygenases (COX), and epoxygenase (CYP P450) give rise to the formation of signal molecules such as eicosanoids, docosanoids and other lipid mediators that participate in intracellular and systemic communication processes mediated by biomolecules such as resolvins, protectins, maresins, and PUFA-derived endocannabinoids [17,18,19,20,21,22,23]. Eicosanoids and docosanoids can activate transmembrane G proteins, stimulating the release of second messengers, such as calcium and cAMP, and interacting with transcription factors (PPAR), which translocate into the nucleus and influence the expression of genes implicated in various cell functions, such as proliferation and inflammation [24,25,26].

Moreover, the by-products of the oxidation of EFAs give rise to well-known molecules, such as prostaglandin, thromboxane, leukotriene, and prostacyclin, which participate in the modulation of various systemic processes such as coagulation and the immune response [27,28].

These bioactive compounds can have a counteracting effect and the two subclasses of EFAs seem to play contrasting roles in these mechanisms. The fatty acids n3 PUFAs have mainly an anti-inflammatory, anticoagulant effect, and reduce the immune response, while n6 PUFAs appear to have pro-inflammatory, immunostimulant effects and promote the coagulation process [15,29,30,31].

However, this division of roles is not so clear-cut, and often specific n6 PUFA molecules can also play anti-inflammatory roles [32]. Gamma linolenic acid (C18:3 cis-6,9,12; GLA), for example, has shown anti-inflammatory effects despite being an essential fatty acid belonging to the n6 PUFA series. It is the precursor of dihomo-GLA acid (C20:3 cis-8,11,14; DGLA), whose oxidation by-product is the anti-inflammatory prostaglandin E1 [33,34]. Certainly, these features are modulated by the concerted effects of the two subclasses of EFAs [35].

**Figure 2:**
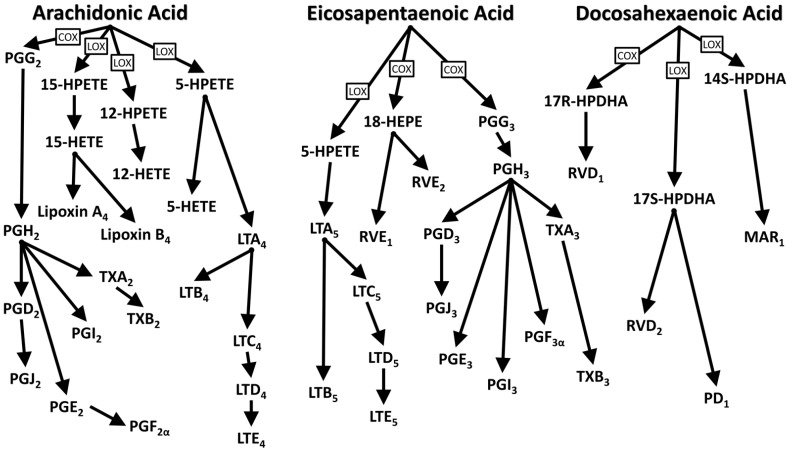
Catabolism of long-chain PUFAs and bioactive molecules formation. Adapted from references [41,42,43,44].

It is interesting to note that the use of n3 LC-PUFAs in the form of supplements is much debated because they are prone to oxidation [36]. The presence of double bonds in the carbonaceous structure exposes them to oxidative degradation and the formation of free radicals [37]. Long-term exposure to oxidized lipids is considered unhealthy [36,38], but at the same time, the signal molecules deriving from EPA and DHA are also by-products of oxidation. Detailed studies that clarify these aspects are still missing. To limit accidental oxidation, a promising solution could derive from nanoencapsulation techniques of PUFAs to protect them from potential degradation factors and favor the attainment of the physiological target [39]. Several micro- and nanoencapsulated formulations of plant-based LC-PUFAs are already commercially available [40]. Figure 2 shows the catabolism of essential fatty acids and derived bioactive molecules.

The oxidative catabolism of DHA, EPA and AA gives rise to biomolecules involved in numerous response mechanisms, such as inflammation, platelet aggregation, immune response and cell proliferation. One of the best-known mechanisms involves TXA_2_ in the initiation process of platelet aggregation. However, AA and EPA compete as substrates for the enzymatic action of COX, and in the presence of adequate amounts of EPA the enzyme is sequestered by the TXA_3_ formation pathway, which does not stimulate vasoconstriction and has a mild pro-aggregating effect [41]. By-products of LC-PUFAs catabolism show contrasting effects that underlie the pleiotropic regulatory mechanism of eicosanoids and docosanoids. A single molecule may have a dual effect, as in the case of PGE_2_, which exhibits a marked pro-inflammatory effect but which may indirectly exert an anti-inflammatory effect by inhibition of the 4-series LT biosynthetic pathway [43]. An imbalance between molecules derived from LC-PUFAs seems to be implicated in some chronic inflammatory pathologies, as in the case of inflammatory bowel disease [45]. Table 2 shows the physiological actions of the main eicosanoids and docosanoids.

### 1.3. Intakes and Requirements of Essential Fatty Acids

#### 1.3.1. Omega-6 to Omega-3 Ratio

The currently available data indicate the requirement of an inadequate intake of n3 LC-PUFAs in human nutrition [46]. As mentioned above, the dietary availability of n6 PUFAs is greater, due to their abundance in the plant kingdom. Our daily requirement of EFAs seems to be biased towards these, with a lower requirement of n3 PUFAs. However, in the diet of industrialized countries, this balance seems to be shifted too much towards n6 PUFAs (15–20:1) due to the reduced dietary sources of n3 PUFAs [47]. In Western countries, this phenomenon could contribute, at least in part, to the greater incidence of inflammatory mechanisms central to several metabolic diseases, such as diabetes, hypertension, autoimmune diseases and chronic inflammatory diseases [48,49,50,51,52,53,54,55,56,57,58,59,60].

Humans could take nutritional advantage of plant dietary sources of n3 PUFA-rich oils extracted from seeds and nuts [61]. However, the choice of the type of sources for lipid production is crucial to obtain an adequate n6:n3 ratio, as seeds and nuts can contain variable proportions of the two subclasses [62]. Although the most appropriate ratio needed between the two classes for human nutrition has not yet been uniquely defined, studies estimated an n6:n3 ratio of about 1–4:1 [63,64,65]. A 4:1 ratio was associated with improvement in secondary prevention, with a 70% reduction in mortality, while a higher ratio of about 2.5:1 seemed to reduce gut cells proliferation among colorectal cancer patients. A ratio of 5:1 was associated with health improvement among patients with asthma. Inflammatory indices were lowered by a 2–3:1 ratio among patients with rheumatoid arthritis [65]. 

#### 1.3.2. Biosynthetic Pathway

**Figure 3:**
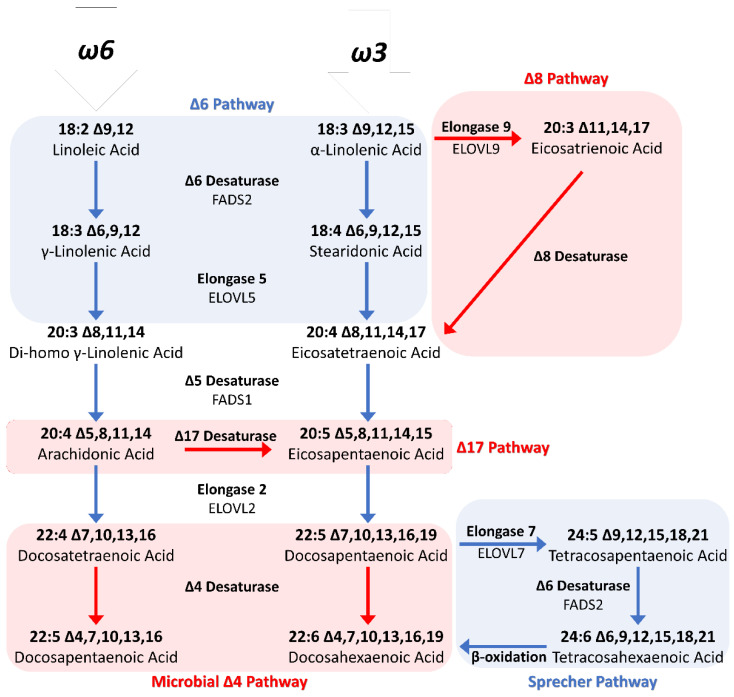
Biosynthesis of essential fatty acids [79,80,81].

The enzymes responsible for the maturation pathway of the precursors are shared for the two subclasses n6 and n3 PUFAs, and this implies that an excess of the former subclass limits the maturation capacity of the latter [66,67]. This means that the conversion of ALA to EPA is affected by the presence of LA, which, in turn, will be converted to AA. Furthermore, even if the precursors can be converted into LC-PUFAs, this activity is very limited by the metabolic efficiency of the enzymatic pull of these metabolic pathways [68,69]. It was estimated that only 0.01–9% of the ALA introduced with the diet can be converted into DHA, with 7–21% converted into EPA [70,71,72,73,74,75,76,77]. Therefore, LC-PUFAs, even if not strictly essential, could be considered conditionally essential [78]. The synthesis of DHA from EPA requires an intermediate elongation step with the biosynthesis of a C24 fatty acid, which is then translocated from the endoplasmic reticulum to the peroxisome to be shortened by beta-oxidation. The biosynthesis of essential fatty acids is shown in Figure 3.

It has not yet been clarified whether this metabolic limit is physiological and therefore describes a narrow need for some specific molecules of LC-PUFAs.

#### 1.3.3. Pregnancy and Lactation: A Programming Window for Neurodevelopment 

Women have a greater ability to convert precursors into LC-PUFAs [70]. This phenomenon, probably caused by the gender difference in specific sex hormones, allows the delivery of enough LC-PUFAs to the fetus and the new born, who both have limited conversion capacities, through placental biomagnification and the mammary gland, respectively [82,83].

Although many studies highlight the efficacy of plant sources in increasing blood concentrations of n3 LC-PUFA and related health benefits, not all studies agree [84,85,86,87,88,89]. Meanwhile, clinical trials investigating the effects of neurocognitive development in offspring using prenatal fish oil (FO) as an intervention have shown inconsistent results [90,91,92,93,94,95,96,97]. In the latest systematic reviews and meta-analyses, infant neurocognitive development showed no differences after LC-PUFAs supplementation during lactation and pregnancy [13,98,99,100,101].

However, as a precautionary principle, at least in some specific stages of life, additional quantities of pre-formed n3 LC-PUFAs are recommended [1,102,103,104,105]. During the development of nervous matter in the last trimester of pregnancy and the first two years of life, the brain preferentially accumulates LC-PUFAs, so AA and DHA make up over 30% of the phospholipid content in the retina and brain [106,107,108,109,110]. However, the ability of the fetus and infants to synthesize LC-PUFAs is insufficient, so they must take advantage of pre-formed EFAs through the placenta and breast milk, respectively [111,112]. Breastfeeding women show blood concentrations of n3 PUFAs that are proportional to those ingested with the diet [113]. Supplementation with algal DHA was effective in increasing the DHA content in breast milk even after premature birth, improving the markers of inflammation [114].

#### 1.3.4. PUFAs Daily Requirements

The global average consumption of n3 LC-PUFAs, estimated from a survey of 266 countries, was 163 mg/day per capita [115]. The WHO recommends 200–500 mg/d of EPA+DHA in adults, and advises reaching adequate amounts of ALA in the case of a vegetarian diet [116]. In the UK, it is recommended to take at least 450 mg/d of EPA+DHA [117]. EFSA, on the other hand, recommends 100 mg/d EPA+DHA for children and 250 mg/d for adults, with an additional amount of 100–200 mg/d of pre-formed DHA during pregnancy and breastfeeding [118], while the IOM recommends an intake of about 1 gram per day of n3 PUFAs, of which at least 10% are LC-PUFAs [119]. The AFSSA recommends 500 mg/d of EPA+DHA during adulthood [120]. The D-A-CH recommends 250 mg/d of EPA+DHA for the general population [121]. The presence of oxidation-prone double bonds raises the doubt that at specific concentrations, EFAs could show toxicity, favoring oxidative stress. However, the most significant concern emerges from using oils rich in PUFAs in a high state of degradation. Nonetheless, the health effect of oxidized products has yet to be adequately explored [36]. The recommendations regarding the intake of essential fatty acids, with particular reference to the n3 LC-PUFAs, are summarized in Table 3.

### 1.4. Availability of Food Sources and Environmental Issues Related to the Supply of Essential Fatty Acids

Based on most common recommendations, the recommended daily intake of n3 LC-PUFA ranges from 250 to 500 mg. If we consider about 7.7 billion people in the world, the global requirement could be between 0.7 and 1.4 million tons of n3 LC-PUFAs per year [69]. 

#### 1.4.1. Fishery

If the demands of the entire population were to be respected, the current availability of marine fish would be insufficient [46]. The nutritional sources of LC-PUFAs for humans are mainly of marine origin [1,124]. However, due to the gradual and continuous depletion of marine species, there is not enough fish to meet the needs of the world population [125,126]. The exploitation of fisheries has reduced the total available fish biomass to 10%, as compared to the pre-industrial era [127]. Furthermore, the rise in the world population will increase the problem of a scarcity of sources [46]. Atlantic and Mediterranean marine resources are increasingly reduced by overfishing [128,129,130,131,132].

#### 1.4.2. Aquaculture

On the other hand, with the use of aquaculture, which has filled the gap of fisheries, the problem of finding sources of LC-PUFAs remains, since feed containing adequate concentrations of LC-PUFAs must be guaranteed to the farmed species [133]. The paradox is that to breed species rich in LC-PUFAs, adequate sources of fishmeal or FO rich in LC-PUFAs must be ensured for aquaculture. These are mainly obtained from marine fisheries, now below sustainable limits and with no prospect of future growth [134]. The current aquaculture practice is to provide feed for fish with added essential amino acids and PUFA-rich vegetables or FO [135]. However, the use in recent years of vegetable oils to replace fishmeal and FO in aquaculture establishments has led to a reduction in EPA/DHA concentrations in farmed animals, highlighting the concept that fish are not the primary producers of n3 LC-PUFAs, and require pre-formed sources of EPA and DHA to incorporate them into their tissues [133].

#### 1.4.3. Safety Concerns

Apart from environmental concerns about aquaculture, there are safety issues to be considered. Due to the bioaccumulation of harmful substances, such as polycyclic aromatics and other persistent and bio-accumulative substances, safety may not be guaranteed [136,137,138,139,140]. Bioaccumulation along the food chains is also a problem in the case of wild fish, and it is well known that sea foods are the main source of methylmercury in human nutrition [138]. These harmful substances accumulate in the adipose tissues and therefore mainly affect the species that should provide LC-PUFAs [141]. For this reason, it was proposed to limit the consumption of these species in at-risk population groups such as children and pregnant women—precisely those individuals who would most benefit from n3 LC-PUFAs [142,143,144].

Moreover, an emerging problem regards the presence of microplastics in the tissues of marine animals. These substances are hard to remove, so they can be ingested with fish-based foods with still unclear consequences [145,146]. Both fisheries and aquaculture show different availability, environmental and health issues, which limit their long-term sustainability. Interestingly, contrary to the bioaccumulation phenomenon of methyl mercury, microplastics seem to accumulate mainly in small fish [147].

#### 1.4.4. Consumer Acceptability

From the consumer’s point of view, fish tends to be less consumed given its cost compared with other foods, the limited acceptance of the fishy taste, and geographical aspects regarding availability [9,148,149]. Some pregnant women do not consume fatty fish as they suffer from hyperemesis and nausea [150,151].

Due to a progressive increase in consumer awareness, more and more individuals also adopt a plant-based diet [152,153]. This implies the abstention from or substantial reduction in meat intake, including fish, with a consequent and involuntary higher intake of omega 6 compared with omega 3 PUFAs [154,155]; however, not all researchers agree [156].

#### 1.4.5. Global Sustainability

In a global vision of sustainability, nutritional sources must be adequate for health, but also be sustainable for the environment and accessible to the ever-growing world population. However, blood concentrations of EPA and DHA are low in the population due to a reduced nutritional intake [157,158]. In such a context, the biotechnology of promising n3 PUFAs plant sources can support the growing demand for EFAs and guarantee an adequate nutritional intake to optimize health needs, without burdening the precarious global resources [159]. The omega-3 fatty acids market is expected to increase globally over the next few years, with a growth rate of 7% over the next 10 years [160]. More and more consumers are searching for omega-3-rich products [161]. This will affect various sectors, including pharmaceuticals, supplements and functional foods [162,163].

#### 1.4.6. Scope

In this regard, this review aims to identify the recent data in the literature on the possible promising plant sources of n3 PUFAs, discuss the technological limitations and the health effects emerging, and suggest future research perspectives that can solve the aforementioned ethical, environmental, and economic issues for global health and sustainability.

## 2. Search Method

The purpose of this narrative review is to highlight the available data on innovative and promising sources of omega-3 fatty acids for human health. To this end, on 27 March 2022 we have conducted a structured search on PubMed (https://pubmed.ncbi.nlm.nih.gov/) by searching the following keywords in titles and abstracts:

(Vegetarian* OR Vegan* OR Vegetable* OR Plant-based* OR Seaweed* OR algal OR algae) AND (Omega-3 OR Eicosapentaenoic OR stearidonic OR docosahexaenoic OR polyunsaturated OR pufa OR epa OR dha OR sda)

Keywords were combined with available MeSH terms. Subsequently, manuscripts written in English covering comparative studies and human clinical studies were selected. Non-human, non-English, book chapters and editorial manuscripts were excluded. Moreover, trials using only animal sources, omega-6 fatty acids or unknown sources of PUFAs were not included.

Bibliography lists from selected manuscripts were checked for further references.

The PubMed search engine was used to determine the completeness of the available database and the efficiency of the filters and Boolean operators.

Even if the search approach was structured, manuscripts were also selected based on relevance to avoid similar trials being discussed.

## 3. Algae

Algae are photosynthetic eukaryotic organisms. There are more than 20,000 species of algae, but their taxonomy is controversial among researchers [164]. Usual groupings do not seem to be animated by phylogenetic aspects and evolutionary lines, but more by the characteristics of organisms with photosynthetic abilities and being aquatic with vegetative structures. A basic classification can be made between microalgae (unicellular algae, simple multicellular or colonial organisms) and macroalgae (or seaweeds) [165].

### 3.1. Macroalgae

Seaweeds, some of which are of food and health interest, can be classified according to the predominant pigments present in them [164,166]:

- Phaeophyta or Brown Algae, including kelp; e.g., Analipus japonicus, Myagropsis myagroides, Padina australis, Sargassum polycytum, Sargassum thunbergii, Ecklonia cava, Ecklonia bicyclis (Arame), Ecklonia stolonifera, Sargassum fusiforme (Hijiki), Undaria pinnatifida (Wakame), Laminaria japonica (Konbu), Laminaria digitata, Laminaria saccharina, Himanthalia elongata (Sea spaghetti), Hizikia fusiforme, Ascophyllum nodosum, Fucus ssp., etc. They owe their color mainly to fucoxanthin;
- Rhodophyta or Red Algae; e.g., Laurencia undulata, Lithothamnion corallioides, Pyropia tenera (Nori), Pyropia yezonensis (Nori), Pyropia umbilicalis (Nori), Chondrus crispus (sea moss), Gracilaria verrucosa, Borentia secundiflora, Palmaria palmata, etc. They contain phycoerythrin, phycocyanin, lutein, zeaxanthin, beta carotene and phycobilin;
- Chlorophyta or Green Algae; e.g., Ulva conglobata, Ulva lactuca (Sea lettuce), Ulva pertusa, Enteromotpha compresa, Caulerpa racemosa, Codium reediae, etc. They owe their color to chlorophyll, lutein, beta carotene, neoxanthin, violaxanthin and zeaxanthin pigments.

There is epidemiological evidence suggesting inverse associations between the consumption of marine algae and chronic degenerative diseases [167]. The benefits of algae can be attributed to numerous compounds contained, such as sterols, terpenes, polysaccharides, carotenoids, tocopherols, fibers, proteins, minerals, vitamins, antioxidants and EFAs [167]. The algae showed a beneficial effect through the regulation of inflammatory-related molecules and enzymes, such as mitogen-activated protein kinases (MAPKs), nuclear factor-kB (NF-kB), inducible nitric oxide synthase (iNOS) and cyclooxygenase (COX-2) [168].

The use of algae for nutraceutical purposes is supported by a consolidated history of use in folk medicine and traditional cuisine, and has become widespread in the West thanks to globalization [169,170]. Human beings have been consuming seaweed for hundreds of years, as evidenced by archeological findings [171]. Furthermore, even if their consumption is related to traditional Asian cuisine with intensive production, their popular use is also traditionally widespread in Europe [172]. The demand for algae as a culinary ingredient continues to grow and it is increasingly considered an attractive food for the consumer [173,174,175]. Furthermore, it can be added to foods thanks to its nutritional characteristics [171].

As mentioned above, humans have a limited ability to convert precursors into LC-PUFAs; however, this limit is also shared by other mammals and many fishes [176,177]. This means that the dietary sources of highly concentrated LC-PUFAs, such as fatty fish, do not owe these concentrations to a greater biosynthetic competence but to accumulation along the trophic chain [135,178,179,180]. At the base of this food web, there are macro- and microalgae [11]. These may have modest concentrations of fats (1.5–4% DW); however, the fraction of EFAs is between 10 and 50% of the total fat content [166]. Algae can synthesize DHA through the delta 6 pathway, the same biosynthetic pathway used by mammals, but also through a direct pathway (delta 4 pathway) that uses a delta 4 desaturase to convert EPA into DHA, or by conversion from n6 PUFAs through delta 17 desaturase action [81]. These aspects are depicted in Figure 3. Biomagnification along the trophic chain is responsible for the high concentrations of LC PUFAs in fatty fish.

Some seaweeds are rich in n3 PUFAs and especially EPA, such as *Analipus japonicusas* (2.6–3.5 mg/g DW), *Sargassum thunbergii* (9–10 mg/g DW) and *Champia parvula* (3.3 mg/g DW) [166]. Rhodophytes can produce high levels of EPA, up to 34% of the algal lipid fraction, and show the best n6:n3 ratio (0.6–1.9). Pheophytes show the highest ratio (2.3–3.9) with extremely variable values in chlorophytes (0.3–31.2) [181]. The highest content of n3 PUFAs was recorded in *Bornetia secundiflora*, a rhodophyte showing 27% of n3 PUFAs and 8% of n6 PUFAs, and therefore with a favorable n6:n3 ratio (0.29). However, the limit of macroalgae is that they contain narrow amounts of lipids [182]. In rhodophytes, the content of n3 PUFAs is around 2.9–27.3%; in phaeophytes it is 6.6–15.4% and in chlorophytes about 9.5–18% [181].

Although the proportions of EFAs concerning the lipid fraction are high, and frequently the content of n3 PUFAs can be higher than n6 PUFAs, the servings consumed through diet are limited to a few grams. This implies that macroalgae as such cannot be considered an effective nutritional source for the supply of LC-PUFAs in human health, representing a negligible source of EFAs in human nutrition that does not exceed 10% of total intakes [135]. On the other hand, the growing demand for cyanobacteria and microalgae such as *Spirulina* and *Chlorella* is due to the fact that they are also considered useful sources of n3 PUFAs [183]. However, taking into account the small fat content, the achievement of adequate EFAs consumption would require the intake of more than 10 grams of dry extracts in powder form, against the generally recommended dosages of a few grams, and therefore can be considered irrelevant to ensure a supply of n3 PUFAs. Additionally, it cannot be underestimated that they contain many other substances and matrix molecules that can influence the effects on health or, on the other hand, can limit the quantities of the product assumable [184,185,186].

However, the biotechnological transformation of macro- and microalgae has shown promising results. Macroalgae contain LC-PUFAs of both the omega 3 and omega 6 subclasses, and the content of EFAs is greater in seaweeds living in cold waters, with EPA representing the main fatty acid [164]. Brown algae often also contain AA in the PUFAs fraction, e.g., *C. crispus* and other species may contain EPA and AA [187], but algal extracts comprising up to 50% of EPA without AA of the lipid fraction can be found, as in the case of *P. palmata* [188].

### 3.2. Microalgae

Regarding microalgae, making up phytoplankton, the classification is more complex and can take into account the size or shape. The inclusion of cyanobacteria, such as *Spirulina* and *Nostoc* (included among the microalgae classification according to the proposed functional classification), is considered by some authors to be improper because they are photosynthetic prokaryotes.

*Chlorella minutissima* can contain from 3 to 31% EPA of the lipid fraction, depending on the strain used, but without any trace of DHA [189]. *Spirulina platensis*, on the other hand, may contain both EPA and DHA in some strains, with lipid fractions ranging from 1.8 to 7.7% [190].

There are potential differences between microalgae and seaweed regarding biotechnological aspects for biomass production. Microalgae may be easier to grow and have fewer variations in fat content, and the fraction proportion of EFAs related to growth conditions changes, such as light exposure [171,191]. They can be farmed in bioreactors, and the production can be scaled up, as in the case of other heterotrophic microorganisms [192]. Furthermore, microalgae can have higher amounts of total fats (20–50% DW) [193]. The temperature is also decisive for unicellular algae, as it has been observed that the highest levels of DHA are obtained in aquatic conditions at lower temperatures [194,195,196]. This aspect has been further discussed concerning the role of these organisms in the food chain, which suggests how a rise in sea temperature could also reduce the presence of n3 LC-PUFAs in marine products and their availability [197]. The environmental impact of the temperature rise could therefore also lead to a reduction in the algal biosynthesis of PUFAs [198]. According to projections for the next 100 years, holding with the current dynamics, the access of the global population to the n3 PUFAs could be reduced to 4% [199].

Two species of microalgae traditionally used as a source of LC-PUFAs are *Schizochytrium* and *Crypthecodinuim* ssp. [135]. Other microalgae candidates for the production of n3 LC-PUFAs are *Monodus subterraneus*, *Odontella aurita*, *Nannochloropsis* ssp., *Pophyridium cruentum* and *Phaeodactylum tricornutum* [200,201]. Many species of microalgae can synthetize only DHA, but it is not so clear if EPA can be obtainable by the endogenous retro conversion of DHA, although recent data have re-evaluated the mechanisms of interconversion of these two molecules [202]. EPA levels of 10.9–12.7% of the lipid fraction were obtained from *Nannochloropis oceanica* and *N. salina* strains, while levels of 22.4–31.4% were obtained from *P. tricornutum* [203,204]. Diatoms have great potential as rich sources of n3 PUFAs, and *P. tricornutum* is one of the diatom species whose genome has been fully sequenced [205]. Although microalgae are a preferential source of DHA, some strains of *Schizochytrium* and *Thraustochytrium* capable of producing both EPA and DHA have been isolated [206]. There is a lot of interest in microalgae species capable of growing in heterotrophic conditions because this greatly facilitates the possibility of reproducing the growing conditions with yields up to 1000 times higher than in autotrophic conditions, and using well-known bio-fermentation systems commonly employed for fungi and bacteria growth [207]. Currently, there are various carbon substrate options for heterotrophic growth, frequently based on by-products from other supply chains such as exhausted olive pomace, birch wood, cane molasses, etc., including volatile fatty acids from waste streams [160]. This is great potential with powerful ecological implications, although these supply chains must be better developed to respect the food-grade characteristics of LC-PUFA production for human use. The species capable of producing PUFAs under heterotrophic conditions include the genera *Schizochytrium* and *Crypthecodinium*, producing DHA [208]. *Thraustrochytrum aureum* strains can produce up to 75% DHA of total fat, while the genus *Ulkenia* reaches up to 13% DHA [209,210].

Oils derived from *Crypthecodinium cohnii* and *Schizochytrium* spp. by heterotrophic growth are already used commercially, especially for the fortification of infant formula and other fortified foods for children [192,211]. The use of plant alternatives in place of FO guarantees greater safety from potential pollutants. Furthermore, since most of the FO produced is used in aquaculture (about 75%), the use of plant oil for the human diet would have a double economic and ecological advantage: reducing the production of FO for human consumption and reducing the use of aquaculture and related costs in the supply chain, intended as a source of PUFAs for humans but also for aquaculture. *Schizochytrium* has been studied as a genus suitable for genetic manipulation to increase the production of n3 LC-PUFAs [212].

As already discussed above, algal oil has been used commercially as a substitute for FO for the formulation of supplements and the fortification of childhood formulas, probably due to its greater safety [213,214,215,216,217,218,219]. It is therefore not surprising that even in the literature, the use of algal extracts is generically considered as concentrated sources of n3 LC-PUFAs. *C. cohnii*, *Ulkenia* sp. and *Schizochytrium* sp. have received Generally Recognized as Safe (GRAS) and Novel Food status for their use as infant and follow-on formula in the USA, Canada and Europe [220,221,222,223,224,225,226,227]. Algal oil supplements are considered safe by the FDA [228].

In an RCT conducted in Mexico, 1094 women received 400 mg/d of algal DHA from 18 or 22 weeks of gestation until delivery to explore the beneficial effects on mothers and offspring from delivery until 5 years of age (ClinicalTrials.gov ID: NCT00646360) [229,230]. The emerging adverse events were not significantly different between the intervention and control groups, and were not related with algal oil intake. Side effects such as nausea, vomiting, and headache were not significantly different between the two groups. The intervention significantly increased the concentrations of DHA in maternal blood at delivery and in cord blood, without significant differences in other PUFAs’ concentrations.

The benefits of LC-PUFAs on offspring neurodevelopment may depend on the presence of specific alleles of the FADS genes encoding delta desaturases, which are prone to single-nucleotide polymorphisms (SNPs). Regarding the presence of FADS gene polymorphisms, 205 pregnant women were randomized to receive 600 mg/d of algal DHA during the last two trimesters of pregnancy (ClinicalTrials.gov ID: NCT00266825) [231,232,233]. Despite the association between the FADS1 genotype and the red blood cells (RBCs) phospholipid DHA concentrations at baseline and in the placebo group at delivery, supplementation with DHA nullified the effect of genetic diversity. However, the FADS2 gene was not associated with baseline and delivery DHA concentrations in either group.

In another RTC on 362 English participants aged 7 to 9 with initial reading difficulties, 600 mg/d of algal DHA (from *Schizochytrium* sp.) was administered for 16 weeks to investigate the effects of n3 LC -PUFAs on sleep-related, cognitive and behavioral aspects (ClinicalTrials.gov ID: NCT01066182) [234,235]. No difference between groups in terms of the incidence of mild adverse events reported emerged, and no side effects were associated with the intervention. The clinical trial was subsequently replicated on 376 children, without reconfirming the positive effects of DHA on neurocognitive, behavioral, and reading skills [236].

Indian people are known to have low intakes of LC-PUFAs [237]. Nine hundred and fifty seven pregnant Indian women were randomized for intervention with 400 mg/d of algal DHA (from *Schizochytrium* sp.), starting from 20 weeks of gestation plus 6 months of lactation (ClinicalTrials.gov ID: NCT03072277) [88,238]. The intervention increased the concentrations of DHA in mothers’ RBCs at delivery and in the cord blood compared to the placebo. No adverse events were related to the treatment. Despite the failure to obtain an improvement in anthropometric, cognitive and neurodevelopment markers in the offspring at 12 months after delivery, the DHA concentrations in children were also significantly higher at 6 months and 12 months compared with placebo.

From a comparative study, 2.4 g/d of LC-PUFA oil from naturally occurring microalgal *Schizochytrum* spp. (DHA:EPA ratio = 2.7:1) was more efficient than 2 g/d FO (DHA:EPA ratio = 0.7:1) in increasing plasma DHA concentrations in a 14 weeks parallel RCT, although FO showed a greater increase in plasma EPA [86]. Both interventions showed comparable effects in reducing TAG concentrations among the 93 hyperglycemic participants.

A 2012 meta-analysis by Bernstein and colleagues, examining 11 RCTs from 1996 to 2011 for a total number of 485 participants, concluded that algal DHA supplements reduced triglyceride concentrations and increased HDL and LDL cholesterol fractions in individuals without cardiovascular disease [239].

In another comparative study, supplementation for 2 weeks with DHA-based algal oil or FO increased plasma DHA concentrations in vegetarian/vegan and omnivore groups [87].

More recently, algal supplements from *Schizochytrium* sp. have been used in clinical trials to evaluate inflammatory outcomes in the treatment of rheumatoid arthritis, cystic fibrosis and bone health, confirming the wide use in the literature of microalgae as sources of n3 LC-PUFAs [240,241,242]. In some cases, supplementation led to an increase in DHA and EPA concentrations from baseline and compared with placebo, indicating not only a direct enrichment of RBCs fatty acid composition, but a possible mechanism of the retro-conversion of DHA to EPA [241,242].

The search for new algal sources of PUFAs is in continuous development, and the identification of new species to be used in large-scale production is decisive for realizing the use of microalgae in the routine of PUFAs supplementation in humans. Microalgae were effective in replacing FO in various health endpoints and enhanced circulating concentrations of PUFAs. It remains to be clarified whether the intake of DHA is sufficient, or whether it is necessary to use strains capable of producing both EPA and DHA [202].

## 4. Other Microorganisms

Some fungi, yeasts and bacteria have been used as single-cell oils due to their ability to accumulate large amounts of intracellular lipids (20–80% of the total biomass) [243]. The *Cunninghamella echinulata* mushroom can produce up to 47% lipids of total biomass [244]. Another alternative to microalgae can be represented by engineered yeasts, such as *Yarrowia lipolytica*, with 50% lipid content of biomass, to obtain an EPA-containing oil for human consumption [245,246]. Unfortunately, even though encapsulated oil formulations have been marketed for human consumption, they represent a source of EPA only [247]. The use of heterotrophic species seems to be more promising, since there is no constraint of light for growth, and this eliminates the main factor that limits cultivation, meaning the grower need only ensure adequate substrate.

The option of being cultivated also on solid substrates and by-products of other supply chains could show the double ecological advantage of their use [248]. The large-scale production of LC-PUFAs has also employed *S. cerevisiae* as a heterologous system for the expression of genes from *Mycobacterium vaccae*, widely used for genetic manipulation, as it is inexpensive, rapid, and has a high safety profile [249]. As for *S. cerevisieae*, *Ashbya gossypii* was also efficiently engineered for the industrial production of PUFAs [250]. Single-cell oils from *Mortierella alpina* and *Pythium* ssp. have been used for the fortification of infant formulas [251]. The processes of scaling up production from small to large scale and extraction remain complex and therefore onerous for obtaining food-grade products [252,253]. Promising species such as *Lipomyces starkeyi*, *Thriosporum pullulans* and *Cryptococcus curvatus* are under study, and can provide potential new biotechnological pathways, but are currently only used in biofuel production [248].

## 5. Plants Rich in Alpha Linolenic Acid

Soy, sunflower and palm are the most used plant sources for the production of food-purpose oils [40]. In the Mediterranean area, extra virgin olive oil is also widely used, which is rich in oleic acid, a monounsaturated fatty acid with well-known health benefits [254]. Palm oil is used as an ingredient in food processing thanks to its thermal stability derived from the high content of saturated fatty acids (40% of palmitic acid) [6]. Soybean oil, on the other hand, contains small fractions of alpha-linolenic acid (about 7%) even if the presence of PUFAs is dominated by LA, which represents over 50% of the total lipid fraction. This implies that soy cannot be considered an adequate source of n3-PUFAs and ALA since the ratio of omega 6 to omega 3 in soybean oil is 10.5:1 [6]. Canola oil is obtained through the selection of specific rapeseed cultivars with a low erucic acid content. It can contain up to 25% of linoleic acid and 9% ALA, with an n6:n3 ratio ranging from 1.9 to 2.5 [255].

Notoriously, some seeds are known to be rich in ALA and are used for the extraction of oil rich in n3 PUFAs. Among these, flaxseeds (*Linum usitatissimum*) have high ALA concentrations with a total fatty acids percentage of 39–60% [4,5].

In a double-blind, crossover RCT, flaxseed oil was shown to significantly increase circulating levels of ALA and EPA in 12 weeks among 15 participants compared to the control (corn oil) [256]. However, DHA levels were not significantly different between the two phases of the trial. Furthermore, the use of flaxseed oil was effective in reducing the levels of small dense LDL-C compared with the corn oil phase. Consistent with these findings, flaxseed oil supplementation was effective in increasing ALA, EPA, and DPA levels, but DHA levels remained unchanged [257]. Supplementation with 10 g of flaxseed oil has also been shown to be effective in reducing circulating free fatty acids and inflammatory marker concentration (tumor necrosis factor alpha) compared with high-oleic sunflower oil, but it was neutral on other vascular risk markers in pre-hypertensive patients [258]. However, the consumption of 15 mL per day of flaxseed oil does not show any additional benefit on inflammatory markers compared to the use of olive oil in young healthy adults [259]. Interestingly, similar metabolic benefits were found after the consumption of fish oil or flaxseed oil [260].

Another plant with seeds rich in ALA is *Salvia hispanica* (chia). Chia seeds can contain up to 64% of ALA of total fatty acids [261,262]. Chia seed has received GRAS status as a source of ALA [263]. The consumption of chia seed oil was effective in transiently increasing DHA levels in human milk following supplementation in the last trimester of pregnancy and the first 3 months of lactation [264].

*Camelina sativa*, a plant belonging to the Brassicaceae family from which oilseeds are obtained, can contain from 19 to 43% of ALA [265]. Similar to what was observed with flaxseed oil, supplementation with Camelina oil favored an increase in circulating ALA concentrations, but without alteration or with a slight reduction in DHA concentrations [266]. However, compared with a fish-rich diet, Camelina oil supplementation was effective in improving the serum lipid profile in individuals with impaired fasting glucose [267].

Elevated levels of ALA can also be found in other plants of regional interest, such as Garden Cress (*Lepedium sativum*), Sacha inchi (*Plukenetia volubilis*), Perilla (*Perilla frutescens*), Basil (*Ocimum basilicum*) and Purslane (*Portulaca oleracea*) [40].

A postprandial trial showed an increase in both ALA and DHA in plasma participants after 2 h from ingestion of Sacha inchi oil, compared with sunflower oil [268].

Purslane and Basil have a marked diffusion in the Mediterranean area, and have ALA lipid fractions above 50% of total fatty acids [5,269,270]. Herbaceous plants show low fat contents (within 4% of dry weight), which do not allow them to be a relevant source of ALA in the case of their consumption as fresh foods. However, Purslane has a favorable n6:n3 ratio of 1:1–3, which could stimulate the use of this specie in the production of concentrated extracts [271,272].

## 6. Plants Rich in Stearidonic Acid

As discussed above, humans and many other vertebrates show a reduced conversion efficiency of ALA into DHA. In addition to the aforementioned mechanism of competition between n3 and n6 PUFAs for the enzyme pool composed of desaturases and elongates that participate in both pathways, there are intrinsic limits that restrict the biosynthesis of LC-PUFAs. Among these, the conversion from EPA to DHA involves some steps of elongation, desaturation, and a subsequent shortening of the aliphatic chain (by the beta-oxidation pathway), which needs translocation into the peroxisome. This pathway, called the “Sprecher Pathway”, is characteristic of vertebrates, but can be bypassed by other pathways in algae (see Section 3.1). Another limiting step is the low affinity of the delta 6 desaturase towards the substrate. This enzyme acts in two steps, the first of which is the conversion of ALA into stearidonic acid (C18: 4 cis-6,9,12,15; SDA), and which directs ALA towards PUFAs maturation in place of lipid catabolism for energy extraction [273]. Furthermore, the two substrates ALA and LA compete for the delta 6 desaturase [274]. The overcoming of this step should facilitate the formation of LC-PUFAs, and so SDA is defined as a pro-eicosapentaenoic acid [275]. The details of the metabolic steps can be seen in Figure 3.

Among the plants, the families of Boraginaceae, Cannabaceae and Primulaceae have a marked delta 6 desaturase activity [276]. Many of these species are wild and have not undergone domestication for agronomic purposes. Hemp oil is extracted from *Cannabis sativa* seeds thanks to their high-fat content, with the final amount of 1–3% of SDA [277]. The use of hemp-based products is booming, but great limitations in the presence of psychoactive substances remain [278].

The use of these plants allows obtaining a source of n3 PUFAs with a more rapid conversion, bypassing one of the most limiting steps of the synthesis pathway of EPA and DHA [279]. The consumption of SDA increases the concentrations of EPA and DPA more than the consumption of ALA [280,281,282,283]. Furthermore, the lower presence of double bonds makes SDA more stable to oxidation than EPA- and DHA-based oils.

The conversion of SDA to EPA appears to be 16–20%, starting from ethyl ester [282,284,285], although the conversion starting from a full-range SDA source can be less efficient [149]. Instead, the conversion of ALA to EPA appears to be 0.2–8% [71,286]. Compared to EPA, SDA could have an efficiency of 0.3:1 for rising EPA concentrations [280].

Among the species of the Boraginaceae family, *Echium plantagineum* and *Lappula patua* seed oil contain about 14% of SDA [287]. Echium oil (EO) in Europe received Novel Food status from the EFSA [288]. Ahiflower (*Buglossoides arvensis*) pertains to the Boraginaceae family and its seed oil may contain up to 16–21% of SDA, with concentrations of ALA of about 50% [279,289,290].

It has been estimated that about 3 g of Ahiflower oil rich in SDA can guarantee 200–250 mg of EPA from the conversion, an amount that would require up to 11 g of ALA from chia or flaxseed oil [289]. The consumption of 11–12 g/d of Ahiflower oil, containing 2.2 g of SDA, is considered GRAS by the American FDA, and a Novel Food by EFSA [291,292]. In an RCT, the intake of 9.1 g/d of Ahiflower oil compared with flaxseed oil showed an increase in EPA and DPA [293]. In a 2017 RCT on 88 healthy subjects, increasing concentrations of Ahiflower oil (0%, 30%, 60% and 100%) were used to assess blood chemistries [294]. Plasma and mononuclear cell EPA and DPA concentrations increased in a dose-dependent fashion, but without changes in DHA concentrations. Interestingly, no clinically significant increase in SDA was observed in the blood after the intake of SDA, suggesting a rapid conversion of the precursor into LC-PUFAs [279].

Recent studies show that *B.arvensis* and *Aegonychon purpurocaeruleum*, both from the Boraginaceae family, display n3 PUFA concentrations of about 80% of the total fatty acids [295]. Ribes, belonging to the Grossulariaceae family, including the well-known blackcurrant (*Ribes Nigrum*), contain about 6% of SDA of total lipids content, and with an n6:n3 ratio of about 1 [296]. Since, especially in Europe, the blackcurrant is used to obtain juices, the by-products containing seeds can be an excellent ecological source of PUFAs [279]. In any case, to obtain adequate amounts of EPA, it is not sufficient that adequate quantities of precursors of n3 PUFAs are provided in the diet, but there is also the need for a correct n6:n3 balance to limit competition with the omega 6 metabolism [75]. Genetics also plays a decisive role as in the case of the FADS genes that encode desaturase enzymes [297]. More active human variants of FADS genes are present at different frequencies in various parts of the world, with less competence in the conversion of ALA to EPA/DHA in Western countries [297]. Epidemiological studies and some clinical trials have shown that EPA and DHA can reduce the risk of cancer, neurological disorders, inflammation and cardiovascular diseases more markedly than ALA and SDA [298,299,300,301]. Although the consumption of SDA does not appear to be effective in increasing DHA, and therefore may have a partial beneficial effect on health, a study on Japanese participants found that rising EPA concentrations, but not DHA, increased cardiovascular protection [302].

EO showed better efficacy than LA-based oil in raising the blood concentrations of EPA and DHA, confirming the greater bioavailability of SDA [85,303]. However, the conversion of SDA to DHA appears to be low [84,85,280], although some authors disagree [282,293,304]. In total, 20 g/d of EO containing 4.8 g of ALA and 1.6 g of SDA for 10 weeks was found to be more effective in increasing erythrocyte EPA than 7.4 g of ALA contained in 20 g of flaxseed oil [85]. Lemke et al. reported that 4.2 g/d SDA increased the omega-3 index (EPA+DHA percentage of total RBCs fatty acids) in a manner comparable to the supplementation of 1 g/d of EPA [282]. Moreover, the intake of 1.5 g/d of SDA can be enough to guarantee the requirement of n3 LC-PUFAs thanks to an EPA conversion efficiency of 17%. Similarly, Harris and colleagues showed that the conversion of ALA to EPA was 0.09%, while the conversion of 6 g/d of SDA to EPA reached 16.6% with an effect equivalent to the supplementation of 1 g/d of EPA [284].

Kuhnt and colleagues conducted a double-blind, parallel-arm clinical trial on 80 individuals randomized to take 17 g/d of EO (containing 5 g of ALA, 2 g of SDA) or FO (containing 1.9 g of EPA and 0.2 g of DHA) for 8 weeks (ClinicalTrials.gov ID: NCT01856179) [84]. The intervention with EO showed an increase in LC-PUFAs in plasma, RBCs and peripheral blood mononuclear cells (PBMCs), with an efficiency of about 25% to 50% compared to FO for EPA and DPA, respectively. However, the intervention showed a decrease in DHA concentrations, despite significant improvements in some metabolic markers such as insulin, TAG, TC, and LDL-C, but also a reduction in HDL-C. The comparator intervention with FO, on the other hand, showed a reduction only in TG and insulin, with an increase in plasma DHA concentration, but this was unchanged in PBMCs. A subgroup of obese individuals or those with metabolic syndrome was recruited into the EO group. BMI was associated with a reduced increase in EPA and DPA. It is well known that the conversion capacity of PUFAs from precursors to LC-PUFA is influenced by various dietary and non-dietary factors [305,306].

From previous work by Kuhnt and colleagues, a more recent and comprehensive double-blind, parallel-arm RCT evaluated the effects of EO (5 g ALA; 2 g SDA) and flaxseed oil (5 g ALA) on 155 individuals for 8 weeks, using olive oil and FO (1.9 g/d EPA; 0.2 g/d DHA) as negative and positive controls, respectively (ClinicalTrials.gov ID: NCT01856179, NCT01217290) [281]. The fatty acid concentrations were evaluated on plasma, RBCs and PBMCs, confirming the increase in EPA and DPA concentrations in the EO group without significant effects for DHA concentrations in the three lipid fractions. EPA and DHA concentrations were greatly increased in the FO group and were unchanged in the OO group, with an EPA efficacy ratio of 100:25:10:0 and DPA ratio of 100:50:25:0 following the intake of FO, EO, flaxseed oil and olive oil, respectively.

Similar to the results above, an RCT with 15 mL/d of echium oil for 7 weeks (containing 3.5 g ALA and 1.4 g SDA) increased EPA concentrations in RBCs by 14% among patients with neck and head cancer in a multicenter, double-blind parallel trial, with null efficacy on improving weight loss (ClinicalTrials.gov ID: NCT01596933) [307]. Unfortunately, DHA concentrations were not assessed.

In a randomized, controlled crossover study on 36 healthy overweight or slightly obese adults, 10 g of EO (containing 2.9 g ALA; 1.2 g SDA) was used as an intervention for 6 weeks compared with high-oleic acid sunflower oil as the control (ClinicalTrials.gov ID: NCT01365078) [308]. The intervention increased the concentrations of EPA and DPA but not DHA in RBCs, without improving the levels of triglycerides or omega-3 index. The efficacy of EO (containing 4.8 g/d ALA; 1.6 g/d SDA) via enriched foods was also compared with algal DHA (1.6 g/d), flaxseed oil (7.4 g/d ALA) and sunflower oil (10 g/d LA) in a double-blind, cross-over RCT for 10 weeks in 49 hyper-triglyceridemic patients (ClinicalTrials.gov ID: NCT01437930) [85]. The concentrations of plasma and RBCs EPA and DPA were significantly higher in the EO phase than in the ALA phase, but there was no significant change in DHA concentrations. Unexpectedly, blood lipids (TC and LDL-C) were improved in all phases of intervention except that with EO. The omega-3 index had increased only in the DHA phase, albeit without a clinically significant increase in the EPA fraction. The reduction in DPA in the phase with algal oil suggests the validity of the DHA to EPA retro-conversion, albeit at levels of little significance from a clinical point of view.

The EO contains both ALA and SDA, and therefore the increases in EPA and DPA concentrations could depend on both precursors. ALA could compete with delta 6 desaturase and inhibit the formation of DHA in the last steps. Furthermore, the presence of GLA, being of the n6 PUFAs subclass, can also contribute by engaging the elongase and desaturase enzymes by making them not available for the maturation of the n3 PUFAs. However, the synthesis of EPA from EO seems comparable with the use of isolated SDA alone [282,309].

Lee and colleagues used a mixture of borage oil and EO (1.9 g/d ALA; 0.83 g/d SDA), compared with positive (FO) and negative (corn oil) controls, in a single-blind, parallel-arms RCT for 8 weeks on 59 individuals with metabolic syndrome or preclinical signs of T2D (ClinicalTrials.gov ID: NCT01145066) [310]. As expected, the combination of plant oils increased serum concentrations of EPA and DPA, but not DHA. The latter was increased only in the positive control group with FO. However, intervention with borage and echium oil significantly reduced total cholesterol and LDL-C concentrations, differently from FO, which reduced triglyceride and increased HDL-C concentrations.

From the most recent literature, it seems clear that SDA is effective in raising the concentrations of EPA and DPA, but not DHA. Recently, a postprandial single-arm clinical trial showed an increase in plasma concentrations of EPA, DPA and DHA, 72 h after a single dose of 26 g EO (7.9 g ALA; 3.1 g SDA) in 12 young and healthy males [303]. While the concentrations of EPA and DHA increased by ~47%, the increase in DHA was 21%. Unfortunately, this was a non-controlled trial involving a limited sample size of only male participants, investigating a single lipid fraction. Although serum lipids were an adequate marker for a postprandial study, the RBCs and PBMC fractions are useful for long-term evaluation and to investigate the influence on the immune system. The DHA concentrations significantly increased at 8 and 72 h, but at 48 h they were lower compared with the baseline. It is also possible that the effect was due to the particularly high intake, as confirmed by the presence of non-serious side effects in half of the participants. Pending further confirmation data, the influence of SDA on DHA is to be considered anecdotal.

Recently, new spontaneous species of Boraginaceae rich in SDA have been identified, many of which are autochthonous of Albania, France, Spain and Italy (mainly in Sicily and Calabria) [295]. Some of these species may contain over 3 g of SDA per 100 g of seeds, and with an n6:n3 ratio of up to 0.3. These cultivars represent excellent candidates for farming and nutraceutical purposes.

## 7. Genetically Modified Plants

Oils obtained from flaxseed, chia, camelina and garden cress are considered ALA-rich oils (19 to 65%) [5,262,265,311]. To find sources of preformed n3 LC-PUFAs without over-exploiting the current already-exhausted supply chains, the progress of genetic engineering can be seen as promising [312]. One of the most consolidated approaches has been the increase in desaturase activity [313]. The genes useful for the biosynthesis of EPA and DHA, present in microalgae, bacteria and yeasts, can be expressed by GM plants to extract oil from their seeds [314]. An example is the engineering of *Arabidopsis thaliana*, a plant belonging to the Brassicaceae family, engineered by the co-expression of various genes that express desaturases (delta 5, delta 8 and delta 9), belonging to *Mortierella alpina*, *Isochrysis galbana* and *Eugenia gracilis* [205,315]. The tobacco plant was also engineered with gene insertion from *Marchantia polymorpha* [316]. The great advantage of this option is that the plants can be easily cultivated while limiting costs, and the production can be scalable thanks to the know-how already available for the cultivation and production of plant oils, represented by the supply chains already used. The big stumbling block in the use of microalgae through cultivation in bioreactors is characterized by production costs [317,318].

From GM plants, it is possible to obtain a broad spectrum of oil seeds containing only EPA, DHA or both, according to the specific biotechnological and nutraceutical needs [319].

Several plants have been used as a source of EFAs, especially seed oils rich in n3 PUFAs. However, it is not yet clear whether the n3 PUFAs precursors can substitute the effect of the n3 LC-PUFAs. A potential role of ALA, apart from its conversion to EPA and DHA, has been proposed for human health [76].

Among the GM plants studied, *Camelina sativa* can be employed to obtain seed oil rich in omega 3, already rich in ALA (28%), and with a good n6:n3 ratio (with an LA concentration of 19%), with good agricultural yields and cheap cultivation [320]. From *C. sativa*, DHA levels similar to those of fish oil can be obtained [321]. The advantage of obtaining crops capable of synthesizing n3 LC-PUFAs can be effective in overcoming the problems of sustainability, acceptability and palatability associated with FO use [153].

There are currently two strains of transgenic crops from *C. sativa*: one that produces EPA and one that produces EPA plus DHA [322,323]. The proportions between EPA and DHA may vary, but a seed oil with 11% EPA and 6% DHA was obtained, very similar to the n3 LC-PUFAs content of FO [314,322].

In a double-blind, cross-over RCT, 36 individuals were randomized for seed oil intake from transgenic *C. sativa* or FO, both containing 450 mg of EPA+DHA (ClinicalTrials.gov ID: NCT03477045). No differences among arms were highlighted at 8 h postprandial regarding the incorporation of the n3 LC-PUFAs into plasma phosphatidylcholine, triacylglycerol or non-esterified fatty acids and cytokine concentrations [324]. The intake of plant oil rich in EPA and DHA caused no clinically significant differences in the increase in plasma (TAG, PC, CE and NEFA) and RBCs concentration of EPA and DHA at 8 weeks, compared with FO in 31 individuals who continued the study after the first postprandial exploratory phase [325]. Similarly, there were no differences in fasting plasma glucose or total blood lipid concentrations for both treatments. No adverse events were associated with the intervention.

These data suggest that plant oil rich in n3 LC-PUFAs from GM plants can be an effective substitute for FO.

GM canola plants are being developed for commercialization. The products obtained will also be destined for human consumption [135]. It was proposed that the use of GM plants may be the most promising approach due to the reduced costs of the supply chains compared to the use of microorganisms in bioreactors [326]. However, in the European Union, there is still strong resistance to the acceptance of GM plants and derived oils due to the perceived safety risk. This opinion is shared by many consumers, and there is also a lack of regulatory directives that can open up their use [327,328].

Soybean, flax and canola were used for genetic bioengineering to produce higher quantities of SDA [309,329,330,331]. For the genes encoding recombinant delta 6 and delta 15 desaturase, the most used sources were *B. officinalis*, *Arabidopsis*, *Phytophthora citrophthora*, *Primula juliae* and *Neurospora crassa* species [331,332].

In a randomized, double-blind, controlled, parallel trial, food containing GM soybean enriched with SDA (7 g/d) was instrumental in the increase in RBC EPA concentration among 50 healthy participants after 12 weeks [333]. No adverse events related to the intervention were observed. However, as expected, SDA did not increase DHA concentrations.

SDA-rich GM soybean has received FDA and EFSA approval [334,335].

The main GM plants and their PUFA compositions are displayed on Table 4.

## 8. Conclusions and Future Remarks

Some algal extracts are now part of the options available for the production of EFAs, considered interchangeable with more traditional fish oils. Certainly, the use of plant options is more environmentally friendly, with fewer hidden risks regarding possible pollutants, and they could show greater acceptability, especially among individuals who have ethically chosen a plant-based diet. The extraction and purification procedures still need to be developed with adequate product standardization. No less important, the choice of the type of supply chain (seaweeds, microalgae or engineered plants) should allow a reduction in production costs for greater access by the world population. Despite a large amount of data being available, although this is beyond the scope of this manuscript, the setting of the clinical studies (such as duration and concentrations of n3 LC-PUFAs) still needs to be standardized to achieve reproducible health outcomes. The inclusion of information about SNPs on FADS genes in the reference population could clarify the discordant data present in the literature. The use of microalgae and other microorganisms in single-cell oil production seems more viable than the extraction from macroalgae, especially for organisms that can be grown in heterotrophic conditions. Macroalgae are very sensitive to the conditions in which they grow, and extraction difficulties are related to the massive presence of other substances such as pigments and fibers, which can limit the extraction and concentration phase of the productions. Heterotrophic microorganisms, on the other hand, can be grown in bioreactors without the need for light. The costs remain equally high for mass production, but future research into the reuse of substrates from by-products of other supply chains could reduce costs. Microalgae are known sources of DHA, but strains capable of providing EPA can also be selected. The choice of plant sources such as plants rich in SDA may lead to obtaining oils that are not effective in stimulating the production of DHA, but only of EPA and DPA. The use of genetically modified plants could overcome the cost-related limits of algal production, providing sources of EPA, DHA or both, if necessary. It is not yet clear whether EPA or DHA alone can be sufficient to satisfy the need for n3 LC-PUFAs. Many species suitable for engineering are easily cultivable and show good yields. There remain some concerns about the use of GM plants, which limits acceptability.

## Figures and Tables

**Table 1:** Concentrations of EFAs in selected plant foods and oils (g per 100 g) ^1^ [7].

Food	18:2 n-6 (LA)	18:3 n-3 (ALA)	18:3 n-6 GLA	18:4 n-3 SDA	20:4 n-6 AA	20:5 n-3 EPA	22:6 n-3 DHA
Almond	12.3	0.003	0	0	0	0	0
Brazil nut	23.9	0.018	0.018	0	0	0	0
Canola oil	18.6	9.14	0	0	0	0	0
Cashew nut	7.78	0.062	NA	0	0	0	0
Chestnut	0.78	0.093	NA	0	0	0	0
Chia seed	5.84	17.8	NA	0	NA	0	0
Coconut oil	1.68	0.019	0	0	0	0	0
Extra virgin Olive oil	8.4	0.65	NA	NA	0	NA	NA
Flaxseed	5.26	19.4	NA	0	NA	0	0
Flaxseed oil	14.2	53.4	0	0	0.015	0	0
hemp seed	27.4	8.68	1.34	0.617	NA	NA	NA
Irish moss	NA	NA	NA	0	NA	0.046	0
Kelp	NA	NA	NA	0.004	NA	0.004	0
Macadamia nut	1.3	0.21	NA	0	0	0	0
Mustard	0.43	0.444	NA	0	0	0	0
Peanut	9.72	0.026	NA	0	0	0	0
Peanut oil	19.7	0.318	NA	0	0.003	0.001	0
Pecan nut	20.6	0.99	NA	0	0	0	0
Pine nut	33.2	0.112	0.052	0	0	0	0
Pistachio	14.1	0.29	NA	0	0	0	0
Pumpkin seed	20.7	0.12	0	0	0	0	0
Sea lettuce	NA	NA	NA	0	NA	0.08	0
Soy nut	NA	NA	NA	0	NA	0	0
Soybean oil	50.9	6.62	0	0	0	0	0
Spirulina	NA	NA	NA	0	NA	0	0
Wakame	NA	NA	NA	0	NA	0.186	0
Walnut	33.8	2.68	0	0	NA	0	0

^1^ NA: Not available.

**Table 2:** Physiological actions of main eicosanoids and docosanoids ([43]).

Action	Biomolecules
Pro-inflammatory	PGE_2_, LTB_4_, LTC_4_
Anti-inflammatory	PGI_2_, RVE_1_, RVD_1_, PD_1_
Pro-aggregatory	PGE_2_, TXA_2_, TXA_3_
Anti-aggregatory	PGI_2_, PGD_2_, PGE_3_, PGI_3_
Immunostimulant	LTB_4_, 12-HETE, Lipoxin A
Immunosuppressive	PGE_3_, Lipoxin B
Vasodilatory	PGE_2_, PGI_2_, PGD_2_
Vasoconstrictor	TXA_2_, PGF_2α_, LTC_4_, LTD_4_, PGE_3_

**Table 3:** Recommended dietary intakes of PUFAs.

Organization	Recommendation/Advice
Joint WHO/FAO 2003 [116]	PUFAs: 6–10% n6 PUFAs: 5–8% n3 PUFAs: 1–2% 200–500 mg/d of EPA+DHA
FAO 2010 [43]	PUFAs: 6–11% n6 PUFAs: 2.5–9% n3 PUFAs: 0.5–2% 250–2000 mg/d of EPA+DHA >300 mg/d of EPA-DHA (pregnancy and lactation) >200 mg/d of DHA (pregnancy and lactation)
SACN 2004 [117]	450 mg/d of n3 LC PUFAs
COMA 1991 [122]	PUFAs: 6% LA: 1% ALA: 0.5%
EFSSA 2010 [118]	LA: 4% ALA: 0.5% 250 mg/d of EPA+DHA 100 mg/d of DHA (6–24 months) Additional 100–200 mg/d of DHA (pregnancy and lactation)
IOM 2005 [119]	n6 PUFAs: 5–10% n3 PUFAs: 0.6–1.2% LC PUFAs: 10% of total n3 and n6 PUFAs, respectively
AFSA 2010 [120]	LA: 4% ALA: 1% 500 mg/d of EPA+DHA (1:1)
D-A-CH 2021 [121]	LA: 2.5% ALA: 0.5% 250 mg/d of EPA+DHA
NNR 2004 [123]	n6 PUFAs: 2.5% n3 PUFAs: 0.5% n6 PUFAs: 4% (pregnancy, lactation and 6–11 months) n3 PUFAs: 1% (pregnancy, lactation and 6–11 months)

**Table 4:** Main characteristic of some GM plants ^1^.

Species	18:2 n6 (LA)	18:3 n3 (ALA)	18:4 n3 (SDA)	20:4 n6 (AA)	20:5 n3 (EPA)	22:6 n3 (DHA)	Reference
*C. sativa*	18.3	13.4	1	2.0	23.1	0	[322]
*C. sativa*	19.2	11.7	3.4	2.4	10.7	6.2	[322]
*B. napus* (canola)	NA	NA	0.26	2.26	7.21	1.02	[336]
*B. napus* (canola)	2–12	4–25	0–4	NA	0–4	6–15	[337]
*N. tabacum*	43.6	29.3	0	1.5	0	NA	[338]
*L. usitatissimum* (flaseed)	5.6	16.8	11.4	1.0	0.8	NA	[338]
*G. max*	15–30	9–12	15–30	NA	NA	NA	[334]
*B. carinata*	4.2	2.0	5.4	5.7	20.4	NA	[339]
*B. juncea*	18.8	6.2	2.2	4.3	5.0	NA	[339]
*A thaliana*	26.0	13.2	0.7	0.4	1.1	2.6	[340]
*A thaliana*	25.9	15.0	1.5	1.0	2.4	5.3	[340]
*A thaliana*	26.4	11.7	1.8	1.6	3.2	0	[340]

^1^ % Total fatty acids. NA: Not available.

## Data Availability

Not applicable.

## Abbreviations

AA	Arachidonic acid
AFSSA	Agence Française de Sécurité Sanitaire des Aliments
ALA	Alpha linolenic acid
BMI	Body mass index
cAMP	Cyclic adenosine monophosphate
CE	Cholesterol ester
COMA	Committee on Medical Aspects
COX	Cyclooxygenase
CYP P450	Cytochrome P450/epoxygenase
DGLA	Dihomo-gamma linolenic acid
DHA	Docosahexaenoic acid
DPA	Docosapentaenoic acid
DW	Dry weight
EFAs	Essential fatty acids
EFSA	European food safety authority
EO	Echium oil
EPA	Eicosapentaenoic acid
FAO	Food and Agriculture Organization
FDA	Food and Drug Administration
FO	Fish oil
GLA	Gamma linolenic acid
GM	Genetically modified
GRAS	Generally recognized as safe
HDL-C	High-density lipoprotein cholesterol
HETE	Hydroxyeicosatetraenoic acid
HPDHA	Hydroperoxydocosahexaenoic acid
HPETE	Hydroperoxyeicosatetraenoic acid
iNOS	Inducible nitric oxide
IOM	Institute of Medicine
LA	Linoleic acid
LC-PUFAs	Long-chain fatty acids
LDL-C	Low-density lipoprotein cholesterol
LOX	Lipoxygenase
LT	Leukotriene
MAPKs	Mitogen-activated protein kinases
MAR	Maresin
NEFA	Not esterified fatty acids
NF-kB	Nuclear factor-kB
NNR	Nordic Nutrition Recommendations
PBMCs	Peripheral blood mononuclear cells
PC	Phosphatidylcholine
PD	Protectin
PG	Prostaglandin
PGI	Prostacyclin
PPAR	Peroxisomal proliferator-activated receptors
PUFAs	Polyunsaturated fatty acids
RBCs	Red blood cells
RCTs	Randomized controlled trials
RV	Resolvin
SACN	Scientific Advisory Committee on Nutrition
SDA	Stearidonic acid
SNPs	Single-nucleotide polymorphisms
TAG	Triacylglycerols
TC	Total cholesterol
TX	Thromboxane
WHO	World Health Organization

## References

[1] Saini R.K., Keum Y.-S. (2018). Omega-3 and Omega-6 Polyunsaturated Fatty Acids: Dietary Sources, Metabolism, and Significance—A Review. Life Sci..

[2] Burr G.O., Burr M.M. (1973). Nutrition Classics from The Journal of Biological Chemistry 82:345-67, 1929. A New Deficiency Disease Produced by the Rigid Exclusion of Fat from the Diet. Nutr. Rev..

[3] (2012). Essential Fatty Acids: The Work of George and Mildred Burr. J. Biol. Chem..

[4] Goyal A., Sharma V., Upadhyay N., Gill S., Sihag M. (2014). Flax and Flaxseed Oil: An Ancient Medicine & Modern Functional Food. J. Food Sci. Technol..

[5] Zamani Ghaleshahi A., Ezzatpanah H., Rajabzadeh G., Ghavami M. (2020). Comparison and Analysis Characteristics of Flax, Perilla and Basil Seed Oils Cultivated in Iran. J. Food Sci. Technol..

[6] Dorni C., Sharma P., Saikia G., Longvah T. (2018). Fatty Acid Profile of Edible Oils and Fats Consumed in India. Food Chem..

[7] Food Data Central. https://fdc.nal.usda.gov/fdc-app.html#/.

[8] Punia S., Sandhu K.S., Siroha A.K., Dhull S.B. (2019). Omega 3-Metabolism, Absorption, Bioavailability and Health Benefits–A Review. PharmaNutrition.

[9] Ian Givens D., Gibbs R.A. (2008). Current Intakes of EPA and DHA in European Populations and the Potential of Animal-Derived Foods to Increase Them. Proc. Nutr. Soc..

[10] Givens D.I., Gibbs R.A. (2006). Very Long Chain N-3 Polyunsaturated Fatty Acids in the Food Chain in the UK and the Potential of Animal-Derived Foods to Increase Intake. Nutr. Bull..

[11] Harwood J.L., Guschina I.A. (2009). The Versatility of Algae and Their Lipid Metabolism. Biochimie.

[12] Innis S.M. (2007). Dietary (n-3) Fatty Acids and Brain Development. J. Nutr..

[13] Campoy C., Escolano-Margarit M.V., Anjos T., Szajewska H., Uauy R. (2012). Omega 3 Fatty Acids on Child Growth, Visual Acuity and Neurodevelopment. Br. J. Nutr..

[14] Mitchell D.C., Niu S.L., Litman B.J. (2001). Optimization of Receptor-G Protein Coupling by Bilayer Lipid Composition I: Kinetics of Rhodopsin-Transducin Binding. J. Biol. Chem..

[15] Layé S., Nadjar A., Joffre C., Bazinet R.P. (2018). Anti-Inflammatory Effects of Omega-3 Fatty Acids in the Brain: Physiological Mechanisms and Relevance to Pharmacology. Pharmacol. Rev..

[16] Rizzo G., Laganà A.S. (2020). The Link between Homocysteine and Omega-3 Polyunsaturated Fatty Acid: Critical Appraisal and Future Directions. Biomolecules.

[17] Calder P.C. (2013). Omega-3 Polyunsaturated Fatty Acids and Inflammatory Processes: Nutrition or Pharmacology?. Br. J. Clin. Pharmacol..

[18] Ortega-Gómez A., Perretti M., Soehnlein O. (2013). Resolution of Inflammation: An Integrated View. EMBO Mol. Med..

[19] Stables M.J., Gilroy D.W. (2011). Old and New Generation Lipid Mediators in Acute Inflammation and Resolution. Prog. Lipid Res..

[20] Astarita G., Kendall A.C., Dennis E.A., Nicolaou A. (2015). Targeted Lipidomic Strategies for Oxygenated Metabolites of Polyunsaturated Fatty Acids. Biochim. Biophys. Acta.

[21] Kim H., Spector A.A. (2018). N-Docosahexaenoylethanolamine: A Neurotrophic and Neuroprotective Metabolite of Docosahexaenoic Acid. Mol. Asp. Med..

[22] Schmitz G., Ecker J. (2008). The Opposing Effects of N−3 and N−6 Fatty Acids. Prog. Lipid Res..

[23] Lands B. (2014). Historical Perspectives on the Impact of N-3 and N-6 Nutrients on Health. Prog. Lipid Res..

[24] Funk C.D. (2001). Prostaglandins and Leukotrienes: Advances in Eicosanoid Biology. Science.

[25] Feige J.N., Gelman L., Michalik L., Desvergne B., Wahli W. (2006). From Molecular Action to Physiological Outputs: Peroxisome Proliferator-Activated Receptors Are Nuclear Receptors at the Crossroads of Key Cellular Functions. Prog. Lipid Res..

[26] Laganà A.S., Vitale S.G., Nigro A., Sofo V., Salmeri F.M., Rossetti P., Rapisarda A.M.C., La Vignera S., Condorelli R.A., Rizzo G. (2016). Pleiotropic Actions of Peroxisome Proliferator-Activated Receptors (PPARs) in Dysregulated Metabolic Homeostasis, Inflammation and Cancer: Current Evidence and Future Perspectives. Int. J. Mol. Sci..

[27] Calder P.C. (2015). Marine Omega-3 Fatty Acids and Inflammatory Processes: Effects, Mechanisms and Clinical Relevance. Biochim. Biophys. Acta.

[28] Weill P., Plissonneau C., Legrand P., Rioux V., Thibault R. (2020). May Omega-3 Fatty Acid Dietary Supplementation Help Reduce Severe Complications in COVID-19 Patients?. Biochimie.

[29] Manuelli M., Della Guardia L., Cena H. (2017). Enriching Diet with N-3 PUFAs to Help Prevent Cardiovascular Diseases in Healthy Adults: Results from Clinical Trials. Int. J. Mol. Sci..

[30] Bäck M. (2017). Omega-3 Fatty Acids in Atherosclerosis and Coronary Artery Disease. Future Sci. OA.

[31] Fischer R., Konkel A., Mehling H., Blossey K., Gapelyuk A., Wessel N., von Schacky C., Dechend R., Muller D.N., Rothe M. (2014). Dietary Omega-3 Fatty Acids Modulate the Eicosanoid Profile in Man Primarily via the CYP-Epoxygenase Pathway. J. Lipid Res..

[32] Johnson G.H., Fritsche K. (2012). Effect of Dietary Linoleic Acid on Markers of Inflammation in Healthy Persons: A Systematic Review of Randomized Controlled Trials. J. Acad. Nutr. Diet..

[33] Mir M. (2008). Echium Oil: A Valuable Source of n-3 and n-6 Fatty Acids. OCL.

[34] Johnson M.M., Swan D.D., Surette M.E., Stegner J., Chilton T., Fonteh A.N., Chilton F.H. (1997). Dietary Supplementation with Gamma-Linolenic Acid Alters Fatty Acid Content and Eicosanoid Production in Healthy Humans. J. Nutr..

[35] Kromhout D., Yasuda S., Geleijnse J.M., Shimokawa H. (2012). Fish Oil and Omega-3 Fatty Acids in Cardiovascular Disease: Do They Really Work?. Eur. Heart J..

[36] Lange K.W., Nakamura Y., Gosslau A.M., Li S. (2019). Are There Serious Adverse Effects of Omega-3 Polyunsaturated Fatty Acid Supplements?. J. Food Bioact..

[37] Nogueira M.S., Scolaro B., Milne G.L., Castro I.A. (2019). Oxidation Products from Omega-3 and Omega-6 Fatty Acids during a Simulated Shelf Life of Edible Oils. LWT.

[38] Zaloga G.P. (2021). Narrative Review of N-3 Polyunsaturated Fatty Acid Supplementation upon Immune Functions, Resolution Molecules and Lipid Peroxidation. Nutrients.

[39] Jurić S., Jurić M., Siddique M.A.B., Fathi M. (2022). Vegetable Oils Rich in Polyunsaturated Fatty Acids: Nanoencapsulation Methods and Stability Enhancement. Food Rev. Int..

[40] Saini R.K., Prasad P., Sreedhar R.V., Akhilender Naidu K., Shang X., Keum Y. (2021). Omega-3 Polyunsaturated Fatty Acids (PUFAs): Emerging Plant and Microbial Sources, Oxidative Stability, Bioavailability, and Health Benefits—A Review. Antioxidants.

[41] Li D. (2011). Chemistry behind Vegetarianism. J. Agric. Food Chem..

[42] Calder P.C., Watson R.R., Preedy V.R. (2013). Chapter 4—Omega-6 and Omega-3 Polyunsaturated Fatty Acids and Inflammatory Bowel Diseases. Bioactive Food as Dietary Interventions for Liver and Gastrointestinal Disease.

[43] Food and Agriculture Organization of the United Nations (2010). Fats and Fatty Acids in Human Nutrition: Report of an Expert Consultation: 10–14 November 2008, Geneva.

[44] Dyall S.C., Balas L., Bazan N.G., Brenna J.T., Chiang N., da Costa Souza F., Dalli J., Durand T., Galano J.-M., Lein P.J. (2022). Polyunsaturated Fatty Acids and Fatty Acid-Derived Lipid Mediators: Recent Advances in the Understanding of Their Biosynthesis, Structures, and Functions. Prog. Lipid Res..

[45] Sharon P., Stenson W.F. (1984). Enhanced Synthesis of Leukotriene B4 by Colonic Mucosa in Inflammatory Bowel Disease. Gastroenterology.

[46] Salem N., Eggersdorfer M. (2015). Is the World Supply of Omega-3 Fatty Acids Adequate for Optimal Human Nutrition?. Curr. Opin. Clin. Nutr. Metab. Care..

[47] Harwood J.L. (2019). Algae: Critical Sources of Very Long-Chain Polyunsaturated Fatty Acids. Biomolecules.

[48] Delgado-Lista J., Perez-Martinez P., Lopez-Miranda J., Perez-Jimenez F. (2012). Long Chain Omega-3 Fatty Acids and Cardiovascular Disease: A Systematic Review. Br. J. Nutr..

[49] Calder P.C. (2014). Very Long Chain Omega-3 (n-3) Fatty Acids and Human Health. Eur. J. Lipid Sci. Technol..

[50] Calder P.C. (2018). Very Long-Chain n-3 Fatty Acids and Human Health: Fact, Fiction and the Future. Proc. Nutr. Soc..

[51] Del Gobbo L.C., Imamura F., Aslibekyan S., Marklund M., Virtanen J.K., Wennberg M., Yakoob M.Y., Chiuve S.E., Dela Cruz L., Frazier-Wood A.C. (2016). ω-3 Polyunsaturated Fatty Acid Biomarkers and Coronary Heart Disease: Pooling Project of 19 Cohort Studies. JAMA Intern. Med..

[52] Mozaffarian D., Lemaitre R.N., King I.B., Song X., Huang H., Sacks F.M., Rimm E.B., Wang M., Siscovick D.S. (2013). Plasma Phospholipid Long-Chain ω-3 Fatty Acids and Total and Cause-Specific Mortality in Older Adults. Ann. Intern. Med..

[53] Simopoulos A.P. (2016). An Increase in the Omega-6/Omega-3 Fatty Acid Ratio Increases the Risk for Obesity. Nutrients.

[54] Grosso G., Pajak A., Marventano S., Castellano S., Galvano F., Bucolo C., Drago F., Caraci F. (2014). Role of Omega-3 Fatty Acids in the Treatment of Depressive Disorders: A Comprehensive Meta-Analysis of Randomized Clinical Trials. PLoS ONE.

[55] Yates C.M., Calder P.C., Ed Rainger G. (2014). Pharmacology and Therapeutics of Omega-3 Polyunsaturated Fatty Acids in Chronic Inflammatory Disease. Pharmacol. Ther..

[56] Wu J.H.Y., Micha R., Imamura F., Pan A., Biggs M.L., Ajaz O., Djousse L., Hu F.B., Mozaffarian D. (2012). Omega-3 Fatty Acids and Incident Type 2 Diabetes: A Systematic Review and Meta-Analysis. Br. J. Nutr..

[57] Chen C., Yu X., Shao S. (2015). Effects of Omega-3 Fatty Acid Supplementation on Glucose Control and Lipid Levels in Type 2 Diabetes: A Meta-Analysis. PLoS ONE.

[58] Sanders T.A.B. (2014). Protective Effects of Dietary PUFA against Chronic Disease: Evidence from Epidemiological Studies and Intervention Trials. Proc. Nutr. Soc..

[59] Griel A.E., Kris-Etherton P.M., Hilpert K.F., Zhao G., West S.G., Corwin R.L. (2007). An Increase in Dietary N-3 Fatty Acids Decreases a Marker of Bone Resorption in Humans. Nutr. J..

[60] Storz M.A. (2020). The Role of Vegan Diets in Lipotoxicity-Induced Beta-Cell Dysfunction in Type-2-Diabetes: A Narrative Review. J. Popul. Ther. Clin. Pharmacol..

[61] Simopoulos A.P. (2002). Omega-3 Fatty Acids in Wild Plants, Nuts and Seeds. Asia Pac. J. Clin. Nutr..

[62] Simopoulos A.P. (2011). Evolutionary Aspects of Diet: The Omega-6/Omega-3 Ratio and the Brain. Mol. Neurobiol..

[63] Molendi-Coste O., Legry V., Leclercq I.A. (2010). Why and How Meet N-3 PUFA Dietary Recommendations?. Gastroenterol. Res. Pract..

[64] Patterson E., Wall R., Fitzgerald G.F., Ross R.P., Stanton C. (2012). Health Implications of High Dietary Omega-6 Polyunsaturated Fatty Acids. J. Nutr. Metab..

[65] Simopoulos A.P. (2002). The Importance of the Ratio of Omega-6/Omega-3 Essential Fatty Acids. Biomed. Pharmacother..

[66] Anderson B.M., Ma D.W.L. (2009). Are All N-3 Polyunsaturated Fatty Acids Created Equal?. Lipids. Health Dis..

[67] Rapoport S.I., Rao J.S., Igarashi M. (2007). Brain Metabolism of Nutritionally Essential Polyunsaturated Fatty Acids Depends on Both the Diet and the Liver. Prostaglandins Leukot Essent Fat. Acids.

[68] Castro L.F.C., Tocher D.R., Monroig O. (2016). Long-Chain Polyunsaturated Fatty Acid Biosynthesis in Chordates: Insights into the Evolution of Fads and Elovl Gene Repertoire. Prog. Lipid Res..

[69] Tocher D.R. (2015). Omega-3 Long-Chain Polyunsaturated Fatty Acids and Aquaculture in Perspective. Aquaculture.

[70] Burdge G.C. (2006). Metabolism of Alpha-Linolenic Acid in Humans. Prostaglandins Leukot Essent Fat. Acids.

[71] Pawlosky R.J., Hibbeln J.R., Novotny J.A., Salem N. (2001). Physiological Compartmental Analysis of Alpha-Linolenic Acid Metabolism in Adult Humans. J. Lipid Res..

[72] Weylandt K.H., Serini S., Chen Y.Q., Su H., Lim K., Cittadini A., Calviello G. (2015). Omega-3 Polyunsaturated Fatty Acids: The Way Forward in Times of Mixed Evidence. BioMed Res. Int..

[73] Burdge G.C., Jones A.E., Wootton S.A. (2002). Eicosapentaenoic and Docosapentaenoic Acids Are the Principal Products of α-Linolenic Acid Metabolism in Young Men. Br. J. Nutr..

[74] Childs C.E., Kew S., Finnegan Y.E., Minihane A.M., Leigh-Firbank E.C., Williams C.M., Calder P.C. (2014). Increased Dietary α-Linolenic Acid Has Sex-Specific Effects upon Eicosapentaenoic Acid Status in Humans: Re-Examination of Data from a Randomised, Placebo-Controlled, Parallel Study. Nutr. J..

[75] Harnack K., Andersen G., Somoza V. (2009). Quantitation of Alpha-Linolenic Acid Elongation to Eicosapentaenoic and Docosahexaenoic Acid as Affected by the Ratio of N6/N3 Fatty Acids. Nutr. Metab..

[76] Plourde M., Cunnane S.C. (2007). Extremely Limited Synthesis of Long Chain Polyunsaturates in Adults: Implications for Their Dietary Essentiality and Use as Supplements. Appl. Physiol. Nutr. Metab..

[77] Brenna J.T., Salem N., Sinclair A.J., Cunnane S.C., International Society for the Study of Fatty Acids and Lipids, ISSFAL (2009). Alpha-Linolenic Acid Supplementation and Conversion to n-3 Long-Chain Polyunsaturated Fatty Acids in Humans. Prostaglandins Leukot Essent Fat. Acids.

[78] Cunnane S.C. (2003). Problems with Essential Fatty Acids: Time for a New Paradigm?. Prog. Lipid Res..

[79] Sayanova O., Napier J.A. (2011). Transgenic Oilseed Crops as an Alternative to Fish Oils. Prostaglandins Leukot. Essent. Fat. Acids.

[80] Meyer A., Kirsch H., Domergue F., Abbadi A., Sperling P., Bauer J., Cirpus P., Zank T.K., Moreau H., Roscoe T.J. (2004). Novel Fatty Acid Elongases and Their Use for the Reconstitution of Docosahexaenoic Acid Biosynthesis. J. Lipid Res..

[81] Delarue J., Guriec N. (2014). Opportunities to Enhance Alternative Sources of Long-Chain n-3 Fatty Acids within the Diet. Proc. Nutr. Soc..

[82] Burdge G. (2004). α-Linolenic Acid Metabolism in Men and Women: Nutritional and Biological Implications. Curr. Opin. Clin. Nutr. Metab. Care.

[83] Welch A.A., Shakya-Shrestha S., Lentjes M.A., Wareham N.J., Khaw K.-T. (2010). Dietary Intake and Status of n–3 Polyunsaturated Fatty Acids in a Population of Fish-Eating and Non-Fish-Eating Meat-Eaters, Vegetarians, and Vegans and the Precursor-Product Ratio of α-Linolenic Acid to Long-Chain n–3 Polyunsaturated Fatty Acids: Results from the EPIC-Norfolk Cohort. Am. J. Clin. Nutr..

[84] Kuhnt K., Fuhrmann C., Köhler M., Kiehntopf M., Jahreis G. (2014). Dietary Echium Oil Increases Long-Chain n–3 PUFAs, Including Docosapentaenoic Acid, in Blood Fractions and Alters Biochemical Markers for Cardiovascular Disease Independently of Age, Sex, and Metabolic Syndrome. J. Nutr..

[85] Dittrich M., Jahreis G., Bothor K., Drechsel C., Kiehntopf M., Blüher M., Dawczynski C. (2015). Benefits of Foods Supplemented with Vegetable Oils Rich in α-Linolenic, Stearidonic or Docosahexaenoic Acid in Hypertriglyceridemic Subjects: A Double-Blind, Randomized, Controlled Trail. Eur. J. Nutr..

[86] Maki K.C., Yurko-Mauro K., Dicklin M.R., Schild A.L., Geohas J.G. (2014). A New, Microalgal DHA- and EPA-Containing Oil Lowers Triacylglycerols in Adults with Mild-to-Moderate Hypertriglyceridemia. Prostaglandins Leukot Essent Fat. Acids.

[87] Ryan L., Symington A.M. (2015). Algal-Oil Supplements Are a Viable Alternative to Fish-Oil Supplements in Terms of Docosahexaenoic Acid (22:6n-3; DHA). J. Funct. Foods.

[88] Khandelwal S., Kondal D., Chaudhry M., Patil K., Swamy M.K., Metgud D., Jogalekar S., Kamate M., Divan G., Gupta R. (2020). Effect of Maternal Docosahexaenoic Acid (DHA) Supplementation on Offspring Neurodevelopment at 12 Months in India: A Randomized Controlled Trial. Nutrients.

[89] Dewell A., Marvasti F.F., Harris W.S., Tsao P., Gardner C.D. (2011). Low- and High-Dose Plant and Marine (n-3) Fatty Acids Do Not Affect Plasma Inflammatory Markers in Adults with Metabolic Syndrome. J. Nutr..

[90] Helland I.B., Saugstad O.D., Smith L., Saarem K., Solvoll K., Ganes T., Drevon C.A. (2001). Similar Effects on Infants of N-3 and n-6 Fatty Acids Supplementation to Pregnant and Lactating Women. Pediatrics.

[91] Helland I.B., Smith L., Saarem K., Saugstad O.D., Drevon C.A. (2003). Maternal Supplementation with Very-Long-Chain n-3 Fatty Acids during Pregnancy and Lactation Augments Children’s IQ at 4 Years of Age. Pediatrics.

[92] Dunstan J.A., Simmer K., Dixon G., Prescott S.L. (2008). Cognitive Assessment of Children at Age 2(1/2) Years after Maternal Fish Oil Supplementation in Pregnancy: A Randomised Controlled Trial. Arch. Dis. Child Fetal. Neonatal Ed..

[93] Makrides M., Gibson R.A., McPhee A.J., Yelland L., Quinlivan J., Ryan P., DOMInO Investigative Team (2010). Effect of DHA Supplementation During Pregnancy on Maternal Depression and Neurodevelopment of Young Children: A Randomized Controlled Trial. JAMA.

[94] Tofail F., Kabir I., Hamadani J.D., Chowdhury F., Yesmin S., Mehreen F., Huda S.N. (2006). Supplementation of Fish-Oil and Soy-Oil during Pregnancy and Psychomotor Development of Infants. J. Health Popul. Nutr..

[95] Smithers L.G., Gibson R.A., McPhee A., Makrides M. (2008). Effect of Long-Chain Polyunsaturated Fatty Acid Supplementation of Preterm Infants on Disease Risk and Neurodevelopment: A Systematic Review of Randomized Controlled Trials. Am. J. Clin. Nutr..

[96] Judge M.P., Harel O., Lammi-Keefe C.J. (2007). A Docosahexaenoic Acid-Functional Food during Pregnancy Benefits Infant Visual Acuity at Four but Not Six Months of Age. Lipids.

[97] Innis S.M., Friesen R.W. (2008). Essential N-3 Fatty Acids in Pregnant Women and Early Visual Acuity Maturation in Term Infants. Am. J. Clin. Nutr..

[98] Gould J.F., Smithers L.G., Makrides M. (2013). The Effect of Maternal Omega-3 (n-3) LCPUFA Supplementation during Pregnancy on Early Childhood Cognitive and Visual Development: A Systematic Review and Meta-Analysis of Randomized Controlled Trials. Am. J. Clin. Nutr..

[99] Dziechciarz P., Horvath A., Szajewska H. (2010). Effects of N-3 Long-Chain Polyunsaturated Fatty Acid Supplementation during Pregnancy and/or Lactation on Neurodevelopment and Visual Function in Children: A Systematic Review of Randomized Controlled Trials. J. Am. Coll. Nutr..

[100] Lo A., Sienna J., Mamak E., Djokanovic N., Westall C., Koren G. (2012). The Effects of Maternal Supplementation of Polyunsaturated Fatty Acids on Visual, Neurobehavioural, and Developmental Outcomes of the Child: A Systematic Review of the Randomized Trials. Obstet. Gynecol. Int..

[101] Larqué E., Gil-Sánchez A., Prieto-Sánchez M.T., Koletzko B. (2012). Omega 3 Fatty Acids, Gestation and Pregnancy Outcomes. Br. J. Nutr..

[102] Aranceta J., Pérez-Rodrigo C. (2012). Recommended Dietary Reference Intakes, Nutritional Goals and Dietary Guidelines for Fat and Fatty Acids: A Systematic Review. Br. J. Nutr..

[103] Calder P.C. (2020). Eicosapentaenoic and Docosahexaenoic Acid Derived Specialised Pro-Resolving Mediators: Concentrations in Humans and the Effects of Age, Sex, Disease and Increased Omega-3 Fatty Acid Intake. Biochimie.

[104] Troesch B., Eggersdorfer M., Laviano A., Rolland Y., Smith A.D., Warnke I., Weimann A., Calder P.C. (2020). Expert Opinion on Benefits of Long-Chain Omega-3 Fatty Acids (DHA and EPA) in Aging and Clinical Nutrition. Nutrients.

[105] Sharma T., Mandal C.C. (2020). Omega-3 Fatty Acids in Pathological Calcification and Bone Health. J. Food Biochem..

[106] Martinez M. (1992). Tissue Levels of Polyunsaturated Fatty Acids during Early Human Development. J. Pediatr..

[107] Lauritzen L., Hansen H.S., Jørgensen M.H., Michaelsen K.F. (2001). The Essentiality of Long Chain N-3 Fatty Acids in Relation to Development and Function of the Brain and Retina. Prog. Lipid Res..

[108] Lauritzen L., Brambilla P., Mazzocchi A., Harsløf L.B.S., Ciappolino V., Agostoni C. (2016). DHA Effects in Brain Development and Function. Nutrients.

[109] Carlson S.E. (2009). Docosahexaenoic Acid Supplementation in Pregnancy and Lactation. Am. J. Clin. Nutr..

[110] Makrides M., Gibson R.A. (2000). Long-Chain Polyunsaturated Fatty Acid Requirements during Pregnancy and Lactation. Am. J. Clin. Nutr..

[111] Koletzko B., Boey C.C.M., Campoy C., Carlson S.E., Chang N., Guillermo-Tuazon M.A., Joshi S., Prell C., Quak S.H., Sjarif D.R. (2014). Current Information and Asian Perspectives on Long-Chain Polyunsaturated Fatty Acids in Pregnancy, Lactation, and Infancy: Systematic Review and Practice Recommendations from an Early Nutrition Academy Workshop. ANM.

[112] Weiser M.J., Butt C.M., Mohajeri M.H. (2016). Docosahexaenoic Acid and Cognition throughout the Lifespan. Nutrients.

[113] Barrera C., Valenzuela R., Chamorro R., Bascuñán K., Sandoval J., Sabag N., Valenzuela F., Valencia M.-P., Puigrredon C., Valenzuela A. (2018). The Impact of Maternal Diet during Pregnancy and Lactation on the Fatty Acid Composition of Erythrocytes and Breast Milk of Chilean Women. Nutrients.

[114] Fougère H., Bilodeau J.-F., Lavoie P.M., Mohamed I., Rudkowska I., Pronovost E., Simonyan D., Berthiaume L., Guillot M., Piedboeuf B. (2021). Docosahexaenoic Acid-Rich Algae Oil Supplementation on Breast Milk Fatty Acid Profile of Mothers Who Delivered Prematurely: A Randomized Clinical Trial. Sci. Rep..

[115] Micha R., Khatibzadeh S., Shi P., Fahimi S., Lim S., Andrews K.G., Engell R.E., Powles J., Ezzati M., Mozaffarian D. (2014). Global, Regional, and National Consumption Levels of Dietary Fats and Oils in 1990 and 2010: A Systematic Analysis Including 266 Country-Specific Nutrition Surveys. BMJ.

[116] World Health Origanization (2003). Diet, Nutrition, and the Prevention of Chronic Diseases: Report of a WHO-FAO Expert Consultation, Geneva, 28 January–1 February 2002.

[117] SACN (2004). Advice on Fish Consumption: Benefits & Risks.

[118] EFSA (2010). EFSA Panel on Dietetic Products, Nutrition, and Allergies (NDA). Scientific Opinion on Dietary Reference Values for Fats, Including Saturated Fatty Acids, Polyunsaturated Fatty Acids, Monounsaturated Fatty Acids, Trans Fatty Acids, and Cholesterol. EFSA J..

[119] Trumbo P., Schlicker S., Yates A., Poos M., Food and Nutrition Board of the Institute of Medicine, The National Academies (2005). Dietary Reference Intakes for Energy, Carbohydrate, Fiber, Fat, Fatty Acids, Cholesterol, Protein, and Amino Acids. J. Am. Diet Assoc..

[120] Opinion of the French Food Safety Agency on the Update of French Population Reference Intakes (ANCs) for Fatty Acids. https://www.anses.fr/en/content/opinion-french-food-safety-agency-update-french-population-reference-intakes-ancs-fatty.

[121] D-A-CH-Referenzwerte für die Nährstoffzufuhr. https://www.dge-medienservice.de/d-a-ch-referenzwerte-fur-die-nahrstoffzufuhr.html.

[122] Britain G., TSO (1991). Dietary Reference Values for Food Energy and Nutrients for the United Kingdom: Report.

[123] (2004). Nordic Nutrition Recommendations. https://www.norden.org/en/publication/nordic-nutrition-recommendations-2004.

[124] Xu H., Turchini G.M., Francis D.S., Liang M., Mock T.S., Rombenso A., Ai Q. (2020). Are Fish What They Eat? A Fatty Acid’s Perspective. Prog. Lipid Res..

[125] FAO (2016). The State of World Fisheries and Aquaculture—2016 (SOFIA): Contributing to Food Security and Nutrition for All.

[126] Worm B., Barbier E.B., Beaumont N., Duffy J.E., Folke C., Halpern B.S., Jackson J.B.C., Lotze H.K., Micheli F., Palumbi S.R. (2006). Impacts of Biodiversity Loss on Ocean Ecosystem Services. Science.

[127] Myers R.A., Worm B. (2003). Rapid Worldwide Depletion of Predatory Fish Communities. Nature.

[128] Hilborn R., Amoroso R.O., Anderson C.M., Baum J.K., Branch T.A., Costello C., de Moor C.L., Faraj A., Hively D., Jensen O.P. (2020). Effective Fisheries Management Instrumental in Improving Fish Stock Status. Proc. Natl. Acad. Sci. USA.

[129] Jackson J.B.C. (2010). The Future of the Oceans Past. Philos. Trans. R. Soc. Biol. Sci..

[130] Hutchings J.A., Reynolds J.D. (2004). Marine Fish Population Collapses: Consequences for Recovery and Extinction Risk. BioScience.

[131] Tocher D. (2009). Issues Surrounding Fish as a Source of Omega-3 Long-Chain Polyunsaturated Fatty Acids. Lipid Technol..

[132] Adarme-Vega T.C., Thomas-Hall S.R., Schenk P.M. (2014). Towards Sustainable Sources for Omega-3 Fatty Acids Production. Curr. Opin. Biotechnol..

[133] Sprague M., Dick J.R., Tocher D.R. (2016). Impact of Sustainable Feeds on Omega-3 Long-Chain Fatty Acid Levels in Farmed Atlantic Salmon, 2006–2015. Sci. Rep..

[134] World Bank (2013). Fish to 2030: Prospects for Fisheries and Aquaculture.

[135] Tocher D.R., Betancor M.B., Sprague M., Olsen R.E., Napier J.A. (2019). Omega-3 Long-Chain Polyunsaturated Fatty Acids, EPA and DHA: Bridging the Gap between Supply and Demand. Nutrients.

[136] Rajeshkumar S., Li X. (2018). Bioaccumulation of Heavy Metals in Fish Species from the Meiliang Bay, Taihu Lake, China. Toxicol. Rep..

[137] Jennings S., Stentiford G.D., Leocadio A.M., Jeffery K.R., Metcalfe J.D., Katsiadaki I., Auchterlonie N.A., Mangi S.C., Pinnegar J.K., Ellis T. (2016). Aquatic Food Security: Insights into Challenges and Solutions from an Analysis of Interactions between Fisheries, Aquaculture, Food Safety, Human Health, Fish and Human Welfare, Economy and Environment. Fish Fish..

[138] Costa L. (2007). Contaminants in Fish: Risk-Benefit Considerations. Arch. Ind. Hyg. Toxicol..

[139] Hites R.A., Foran J.A., Carpenter D.O., Hamilton M.C., Knuth B.A., Schwager S.J. (2004). Global Assessment of Organic Contaminants in Farmed Salmon. Science.

[140] Foran J.A., Carpenter D.O., Hamilton M.C., Knuth B.A., Schwager S.J. (2005). Risk-Based Consumption Advice for Farmed Atlantic and Wild Pacific Salmon Contaminated with Dioxins and Dioxin-like Compounds. Environ. Health Perspect..

[141] Rizzo G., Baroni L. (2016). Health and Ecological Implications of Fish Consumption: A Deeper Insight. Mediterr. J. Nutr. Metab..

[142] EFSA Scientific Opinion on the Risk for Public Health Related to the Presence of Mercury and Methylmercury in Food. https://www.efsa.europa.eu/en/efsajournal/pub/2985.

[143] Commission Regulation (EC) No. 1881/2006 Setting Maximum Levels for Certain Contaminants in Foodstuffs.|UNEP Law and Environment Assistance Platform. https://leap.unep.org/countries/eu/national-legislation/commission-regulation-ec-no-18812006-setting-maximum-levels.

[144] Storelli M.M., Stuffler R.G., Marcotrigiano G.O. (2002). Total and Methylmercury Residues in Tuna-Fish from the Mediterranean Sea. Food Addit. Contam..

[145] Smith M., Love D.C., Rochman C.M., Neff R.A. (2018). Microplastics in Seafood and the Implications for Human Health. Curr. Environ. Health Rep..

[146] Salerno M., Berlino M., Mangano M.C., Sarà G. (2021). Microplastics and the Functional Traits of Fishes: A Global Meta-Analysis. Glob. Chang. Biol..

[147] Pennino M.G., Bachiller E., Lloret-Lloret E., Albo-Puigserver M., Esteban A., Jadaud A., Bellido J.M., Coll M. (2020). Ingestion of Microplastics and Occurrence of Parasite Association in Mediterranean Anchovy and Sardine. Mar. Pollut. Bull..

[148] Baker E.J., Miles E.A., Burdge G.C., Yaqoob P., Calder P.C. (2016). Metabolism and Functional Effects of Plant-Derived Omega-3 Fatty Acids in Humans. Prog. Lipid Res..

[149] Maki K.C., Rains T.M. (2012). Stearidonic Acid Raises Red Blood Cell Membrane Eicosapentaenoic Acid. J. Nutr..

[150] Chan R.L., Olshan A.F., Savitz D.A., Herring A.H., Daniels J.L., Peterson H.B., Martin S.L. (2011). Maternal Influences on Nausea and Vomiting in Early Pregnancy. Matern. Child Health J..

[151] McCarthy F.P., Lutomski J.E., Greene R.A. (2014). Hyperemesis Gravidarum: Current Perspectives. Int. J. Womens Health.

[152] Rosell M.S., Lloyd-Wright Z., Appleby P.N., Sanders T.A.B., Allen N.E., Key T.J. (2005). Long-Chain n-3 Polyunsaturated Fatty Acids in Plasma in British Meat-Eating, Vegetarian, and Vegan Men. Am. J. Clin. Nutr..

[153] Burdge G.C., Tan S., Henry C.J. (2017). Long-Chain n-3 PUFA in Vegetarian Women: A Metabolic Perspective. J. Nutr. Sci..

[154] Rogerson D. (2017). Vegan Diets: Practical Advice for Athletes and Exercisers. J. Int. Soc. Sport. Nutr..

[155] Clarys P., Deliens T., Huybrechts I., Deriemaeker P., Vanaelst B., De Keyzer W., Hebbelinck M., Mullie P. (2014). Comparison of Nutritional Quality of the Vegan, Vegetarian, Semi-Vegetarian, Pesco-Vegetarian and Omnivorous Diet. Nutrients.

[156] Kahleova H., Hlozkova A., Fleeman R., Fletcher K., Holubkov R., Barnard N.D. (2019). Fat Quantity and Quality, as Part of a Low-Fat, Vegan Diet, Are Associated with Changes in Body Composition, Insulin Resistance, and Insulin Secretion. A 16-Week Randomized Controlled Trial. Nutrients.

[157] Kris-Etherton P.M., Harris W.S., Appel L.J., American Heart Association (2002). Nutrition Committee Fish Consumption, Fish Oil, Omega-3 Fatty Acids, and Cardiovascular Disease. Circulation.

[158] Stark K.D., Van Elswyk M.E., Higgins M.R., Weatherford C.A., Salem N. (2016). Global Survey of the Omega-3 Fatty Acids, Docosahexaenoic Acid and Eicosapentaenoic Acid in the Blood Stream of Healthy Adults. Prog. Lipid Res..

[159] Sprague M., Betancor M.B., Tocher D.R. (2017). Microbial and Genetically Engineered Oils as Replacements for Fish Oil in Aquaculture Feeds. Biotechnol. Lett..

[160] Oliver L., Dietrich T., Marañón I., Villarán M.C., Barrio R.J. (2020). Producing Omega-3 Polyunsaturated Fatty Acids: A Review of Sustainable Sources and Future Trends for the EPA and DHA Market. Resources.

[161] Omega-3: Global Product Trends and Opportunities: Market Research Report. https://www.packagedfacts.com/Omega-Global-Product-6385341/.

[162] Omega 3 Market Size & Share Report, 2020–2028. https://www.grandviewresearch.com/industry-analysis/omega-3-market.

[163] Omega-3 Market by Type (DHA, EPA, and ALA), Application (Dietary Supplements, Functional Foods & Beverages, Pharmaceuticals, Infant Formula, and Pet Food & Feed), Source (Marine and Plant), and Region—Global Forecasts to 2025. https://www.marketresearch.com/MarketsandMarkets-v3719/Omega-Type-DHA-EPA-ALA-12837793/.

[164] Barbalace M.C., Malaguti M., Giusti L., Lucacchini A., Hrelia S., Angeloni C. (2019). Anti-Inflammatory Activities of Marine Algae in Neurodegenerative Diseases. Int. J. Mol. Sci..

[165] Blunt J.W., Carroll A.R., Copp B.R., Davis R.A., Keyzers R.A., Prinsep M.R. (2018). Marine Natural Products. Nat. Prod. Rep..

[166] Bocanegra A., Macho-González A., Garcimartín A., Benedí J., Sánchez-Muniz F.J. (2021). Whole Alga, Algal Extracts, and Compounds as Ingredients of Functional Foods: Composition and Action Mechanism Relationships in the Prevention and Treatment of Type-2 Diabetes Mellitus. Int. J. Mol. Sci..

[167] Brown E.S., Allsopp P.J., Magee P.J., Gill C.I.R., Nitecki S., Strain C.R., McSorley E.M. (2014). Seaweed and Human Health. Nutr. Rev..

[168] Yu D.-K., Lee B., Kwon M., Yoon N., Shin T., Kim N.-G., Choi J.-S., Kim H.-R. (2015). Phlorofucofuroeckol B Suppresses Inflammatory Responses by Down-Regulating Nuclear Factor ΚB Activation via Akt, ERK, and JNK in LPS-Stimulated Microglial Cells. Int. Immunopharmacol..

[169] Koyande A.K., Chew K.W., Rambabu K., Tao Y., Chu D.-T., Show P.-L. (2019). Microalgae: A Potential Alternative to Health Supplementation for Humans. Food Sci. Hum. Wellness.

[170] Hannon B.A., Fairfield W.D., Adams B., Kyle T., Crow M., Thomas D.M. (2020). Use and Abuse of Dietary Supplements in Persons with Diabetes. Nutr. Diabetes.

[171] Wells M.L., Potin P., Craigie J.S., Raven J.A., Merchant S.S., Helliwell K.E., Smith A.G., Camire M.E., Brawley S.H. (2017). Algae as Nutritional and Functional Food Sources: Revisiting Our Understanding. J. Appl. Phycol..

[172] Analisis de Especies, European Commission (2019). Directorate General for Maritime Affairs and Fisheries.

[173] Afonso N.C., Catarino M.D., Silva A.M.S., Cardoso S.M. (2019). Brown Macroalgae as Valuable Food Ingredients. Antioxidants.

[174] Khan M.I., Shin J.H., Kim J.D. (2018). The Promising Future of Microalgae: Current Status, Challenges, and Optimization of a Sustainable and Renewable Industry for Biofuels, Feed, and Other Products. Microb. Cell Factories.

[175] Kent M., Welladsen H.M., Mangott A., Li Y. (2015). Nutritional Evaluation of Australian Microalgae as Potential Human Health Supplements. PLoS ONE.

[176] Gladyshev M.I., Sushchik N.N. (2019). Long-Chain Omega-3 Polyunsaturated Fatty Acids in Natural Ecosystems and the Human Diet: Assumptions and Challenges. Biomolecules.

[177] Twining C.W., Brenna J.T., Hairston N.G., Flecker A.S. (2016). Highly Unsaturated Fatty Acids in Nature: What We Know and What We Need to Learn. Oikos.

[178] Kabeya N., Fonseca M.M., Ferrier D.E.K., Navarro J.C., Bay L.K., Francis D.S., Tocher D.R., Castro L.F.C., Monroig Ó. (2018). Genes for de Novo Biosynthesis of Omega-3 Polyunsaturated Fatty Acids Are Widespread in Animals. Sci. Adv..

[179] Camacho-Rodríguez J., Macías-Sánchez M.D., Cerón-García M.C., Alarcón F.J., Molina-Grima E. (2018). Microalgae as a Potential Ingredient for Partial Fish Meal Replacement in Aquafeeds: Nutrient Stability under Different Storage Conditions. J. Appl. Phycol..

[180] Dineshbabu G., Goswami G., Kumar R., Sinha A., Das D. (2019). Microalgae–Nutritious, Sustainable Aqua- and Animal Feed Source. J. Funct. Food.

[181] Pereira H., Barreira L., Figueiredo F., Custódio L., Vizetto-Duarte C., Polo C., Rešek E., Engelen A., Varela J. (2012). Polyunsaturated Fatty Acids of Marine Macroalgae: Potential for Nutritional and Pharmaceutical Applications. Mar. Drugs.

[182] Maehre H.K., Malde M.K., Eilertsen K.-E., Elvevoll E.O. (2014). Characterization of Protein, Lipid and Mineral Contents in Common Norwegian Seaweeds and Evaluation of Their Potential as Food and Feed. J. Sci. Food Agric..

[183] Andrade L.M., Andrade C.J., Dias M., Nascimento C.A., Mendez M.A. (2018). Chlorella and Spirulina Microalgae as Sources of Functional Foods, Nutraceuticals, and Food Supplements; an Overview. MOJ Food Process. Technol..

[184] Lee S.H., Kang H.J., Lee H., Kang M., Park Y.K. (2010). Six-Week Supplementation with Chlorella Has Favorable Impact on Antioxidant Status in Korean Male Smokers. Nutrition.

[185] Deng R., Chow T.J. (2010). Hypolipidemic, Antioxidant, and Antiinflammatory Activities of Microalgae Spirulina. Cardiovasc. Ther..

[186] Dartsch P.C. (2008). Antioxidant Potential of Selected Spirulina Platensis Preparations. Phytother. Res..

[187] Fleurence J., Gutbier G., Mabeau S., Leray C. (1994). Fatty Acids from 11 Marine Macroalgae of the French Brittany Coast. J. Appl. Phycol..

[188] van Ginneken V.J., Helsper J.P., de Visser W., van Keulen H., Brandenburg W.A. (2011). Polyunsaturated Fatty Acids in Various Macroalgal Species from North Atlantic and Tropical Seas. Lipids Health Dis..

[189] Vazhappilly R., Chen F. (1998). Eicosapentaenoic Acid and Docosahexaenoic Acid Production Potential of Microalgae and Their Heterotrophic Growth. J. Am. Oil Chem. Soc..

[190] Diraman H., Koru E., Dibeklioglu H. (2009). Fatty Acid Profile of Spirulina Platensis Used as a Food Supplement. Isr. J. Aquac..

[191] Bocanegra A., Bastida S., Benedí J., Ródenas S., Sánchez-Muniz F.J. (2009). Characteristics and Nutritional and Cardiovascular-Health Properties of Seaweeds. J. Med. Food.

[192] Winwood R.J. (2013). Recent Developments in the Commercial Production of DHA and EPA Rich Oils from Micro-Algae. OCL.

[193] Barkia I., Saari N., Manning S.R. (2019). Microalgae for High-Value Products Towards Human Health and Nutrition. Mar. Drugs.

[194] Renaud S.M., Thinh L.V., Lambrinidis G., Parry D.L. (2002). Effect of Temperature on Growth, Chemical Composition and Fatty Acid Composition of Tropical Australian Microalgae Grown in Batch Cultures. Aquaculture.

[195] Ackman R.G., Tocher C.S., McLachlan J. (1968). Marine Phytoplankter Fatty Acids. J. Fish Res. Bd. Can..

[196] Hixson S.M., Arts M.T. (2016). Climate Warming Is Predicted to Reduce Omega-3, Long-Chain, Polyunsaturated Fatty Acid Production in Phytoplankton. Glob. Chang. Biol..

[197] Colombo S.M., Rodgers T.F.M., Diamond M.L., Bazinet R.P., Arts M.T. (2020). Projected Declines in Global DHA Availability for Human Consumption as a Result of Global Warming. Ambio.

[198] Fuschino J.R., Guschina I.A., Dobson G., Yan N.D., Harwood J.L., Arts M.T. (2011). Rising Water Temperatures Alter Lipid Dynamics and Reduce N-3 Essential Fatty Acid Concentrations in Scenedesmus Obliquus (Chlorophyta)1. J. Phycol..

[199] Seong T., Matsutani H., Haga Y., Kitagima R., Satoh S. (2019). First Step of Non-Fish Meal, Non-Fish Oil Diet Development for Red Seabream, (*Pagrus major*), with Plant Protein Sources and Microalgae *Schizochytrium* sp.. Aquac. Res..

[200] Bernaerts T.M.M., Gheysen L., Kyomugasho C., Jamsazzadeh Kermani Z., Vandionant S., Foubert I., Hendrickx M.E., Van Loey A.M. (2018). Comparison of Microalgal Biomasses as Functional Food Ingredients: Focus on the Composition of Cell Wall Related Polysaccharides. Algal Res..

[201] Ma Y., Wang Z., Yu C., Yin Y., Zhou G. (2014). Evaluation of the Potential of 9 Nannochloropsis Strains for Biodiesel Production. Bioresour. Technol..

[202] Metherel A.H., Bazinet R.P. (2019). Updates to the N-3 Polyunsaturated Fatty Acid Biosynthesis Pathway: DHA Synthesis Rates, Tetracosahexaenoic Acid and (Minimal) Retroconversion. Prog. Lipid Res..

[203] Ryckebosch E., Bruneel C., Termote-Verhalle R., Goiris K., Muylaert K., Foubert I. (2014). Nutritional Evaluation of Microalgae Oils Rich in Omega-3 Long Chain Polyunsaturated Fatty Acids as an Alternative for Fish Oil. Food Chem..

[204] Hamilton M.L., Warwick J., Terry A., Allen M.J., Napier J.A., Sayanova O. (2015). Towards the Industrial Production of Omega-3 Long Chain Polyunsaturated Fatty Acids from a Genetically Modified Diatom Phaeodactylum Tricornutum. PLoS ONE.

[205] Barclay W.R., Meager K.M., Abril J.R. (1994). Heterotrophic Production of Long Chain Omega-3 Fatty Acids Utilizing Algae and Algae-like Microorganisms. J. Appl. Phycol..

[206] Adarme-Vega T.C., Lim D.K.Y., Timmins M., Vernen F., Li Y., Schenk P.M. (2012). Microalgal Biofactories: A Promising Approach towards Sustainable Omega-3 Fatty Acid Production. Microb. Cell Fact..

[207] Byreddy A.R. (2016). Thraustochytrids as an Alternative Source of Omega-3 Fatty Acids, Carotenoids and Enzymes. Lipid Technol..

[208] Gupta A., Barrow C.J., Puri M. (2012). Omega-3 Biotechnology: Thraustochytrids as a Novel Source of Omega-3 Oils. Biotechnol. Adv..

[209] Lee Chang K.J., Nichols C.M., Blackburn S.I., Dunstan G.A., Koutoulis A., Nichols P.D. (2014). Comparison of Thraustochytrids *Aurantiochytrium* Sp., *Schizochytrium* Sp., *Thraustochytrium* Sp., and *Ulkenia* Sp. for Production of Biodiesel, Long-Chain Omega-3 Oils, and Exopolysaccharide. Mar. Biotechnol..

[210] Chalima A., Oliver L., Fernández de Castro L., Karnaouri A., Dietrich T., Topakas E. (2017). Utilization of Volatile Fatty Acids from Microalgae for the Production of High Added Value Compounds. Fermentation.

[211] Yeiser M., Harris C.L., Kirchoff A.L., Patterson A.C., Wampler J.L., Zissman E.N., Berseth C.L. (2016). Growth and Tolerance of Infants Fed Formula with a New Algal Source of Docosahexaenoic Acid: Double-Blind, Randomized, Controlled Trial. Prostaglandins Leukot Essent Fat. Acids.

[212] Mühlroth A., Li K., Røkke G., Winge P., Olsen Y., Hohmann-Marriott M.F., Vadstein O., Bones A.M. (2013). Pathways of Lipid Metabolism in Marine Algae, Co-Expression Network, Bottlenecks and Candidate Genes for Enhanced Production of EPA and DHA in Species of Chromista. Mar. Drugs.

[213] Hoffman D.R., Wheaton D.K.H., James K.J., Tuazon M., Diersen-Schade D.A., Harris C.L., Stolz S., Berseth C.L. (2006). Docosahexaenoic Acid in Red Blood Cells of Term Infants Receiving Two Levels of Long-Chain Polyunsaturated Fatty Acids. J. Pediatr. Gastroenterol. Nutr..

[214] Hoffman D.R., Birch E.E., Birch D.G., Uauy R., Castañeda Y.S., Lapus M.G., Wheaton D.H. (2000). Impact of Early Dietary Intake and Blood Lipid Composition of Long-Chain Polyunsaturated Fatty Acids on Later Visual Development. J. Pediatr. Gastroenterol. Nutr..

[215] Birch E.E., Hoffman D.R., Uauy R., Birch D.G., Prestidge C. (1998). Visual Acuity and the Essentiality of Docosahexaenoic Acid and Arachidonic Acid in the Diet of Term Infants. Pediatr. Res..

[216] Birch E.E., Castañeda Y.S., Wheaton D.H., Birch D.G., Uauy R.D., Hoffman D.R. (2005). Visual Maturation of Term Infants Fed Long-Chain Polyunsaturated Fatty Acid-Supplemented or Control Formula for 12 Mo. Am. J. Clin. Nutr..

[217] Innis S.M., Adamkin D.H., Hall R.T., Kalhan S.C., Lair C., Lim M., Stevens D.C., Twist P.F., Diersen-Schade D.A., Harris C.L. (2002). Docosahexaenoic Acid and Arachidonic Acid Enhance Growth with No Adverse Effects in Preterm Infants Fed Formula. J. Pediatr..

[218] Clandinin M.T., Van Aerde J.E., Merkel K.L., Harris C.L., Springer M.A., Hansen J.W., Diersen-Schade D.A. (2005). Growth and Development of Preterm Infants Fed Infant Formulas Containing Docosahexaenoic Acid and Arachidonic Acid. J. Pediatr..

[219] Hoffman D., Ziegler E., Mitmesser S.H., Harris C.L., Diersen-Schade D.A. (2008). Soy-Based Infant Formula Supplemented with DHA and ARA Supports Growth and Increases Circulating Levels of These Fatty Acids in Infants. Lipids.

[220] GRAS Notices GRN No. 844 Algal Oil (55% Docosahexaenoic Acid) from *Schizochytrium* Sp. Strain FCC-3204. https://www.cfsanappsexternal.fda.gov/scripts/fdcc/?set=GRASNotices&id=844&sort=GRN_No&order=DESC&startrow=1&type=basic&search=844.

[221] GRAS Notices GRN No. 677 Docosahexaenoic Acid Oil Produced in *Schizochytrium* sp.. https://www.cfsanappsexternal.fda.gov/scripts/fdcc/?set=GRASNotices&id=677&sort=GRN_No&order=DESC&startrow=1&type=basic&search=677.

[222] GRAS Notices GRN No. 860 Algal Oil (36% Docosahexaenoic Acid) from *Schizochytrium* Sp. Strain DHF. https://www.cfsanappsexternal.fda.gov/scripts/fdcc/?set=GRASNotices&id=860&sort=GRN_No&order=DESC&startrow=1&type=basic&search=860.

[223] GRAS Notices GRN No. 553 Algal Oil (40% Docosahexaenoic Acid) Derived from *Schizochytrium* sp.. https://www.cfsanappsexternal.fda.gov/scripts/fdcc/?set=GRASNotices&id=553&sort=GRN_No&order=DESC&startrow=1&type=basic&search=553.

[224] Safety of *Schizochytrium* Sp. Oil as a Novel Food Pursuant to Regulation (EU) 2015/2283|EFSA. https://www.efsa.europa.eu/en/efsajournal/pub/6242.

[225] Canada H. List of Non-Novel Determinations for Food and Food Ingredients. https://www.canada.ca/en/health-canada/services/food-nutrition/genetically-modified-foods-other-novel-foods/requesting-novelty-determination/list-non-novel-determinations.html.

[226] (2015). Commission Implementing Decision (EU) 2015/545 of 31 March 2015 Authorising the Placing on the Market of Oil from the Micro-Algae *Schizochytrium* sp. (ATCC PTA-9695) as a Novel Food Ingredient under Regulation (EC) No 258/97 of the European Parliament and of the Council (Notified under Document C(2015) 2082); Official Journal of the European Union, European Union. https://faolex.fao.org/docs/pdf/eur142925.pdf.

[227] (2015). Commission Delegated Regulation (EU) 2016/127 of 25 September 2015 Supplementing Regulation (EU) No 609/2013 of the European Parliament and of the Council as Regards the Specific Compositional and Information Requirements for Infant Formula and Follow-on Formula and as Regards Requirements on Information Relating to Infant and Young Child Feeding (Text with EEA Relevance); Official Journal of the European Union, European Union. https://faolex.fao.org/docs/pdf/eur151710.pdf..

[228] GRAS Notices GRN No. 732 Docosahexaenoic Acid Oil Produced in *Schizochytrium* sp.. https://www.cfsanappsexternal.fda.gov/scripts/fdcc/?set=GRASNotices&id=732&sort=GRN_No&order=DESC&startrow=1&type=basic&search=732.

[229] Gonzalez Casanova I., Schoen M., Tandon S., Stein A.D., Barraza Villarreal A., DiGirolamo A.M., Demmelmair H., Ramirez Silva I., Feregrino R.G., Rzehak P. (2021). Maternal FADS2 Single Nucleotide Polymorphism Modified the Impact of Prenatal Docosahexaenoic Acid (DHA) Supplementation on Child Neurodevelopment at 5 Years: Follow-up of a Randomized Clinical Trial. Clin. Nutr..

[230] Ramakrishnan U., Stein A.D., Parra-Cabrera S., Wang M., Imhoff-Kunsch B., Juárez-Márquez S., Rivera J., Martorell R. (2010). Effects of Docosahexaenoic Acid Supplementation during Pregnancy on Gestational Age and Size at Birth: Randomized, Double-Blind, Placebo-Controlled Trial in Mexico. Food Nutr. Bull..

[231] Carlson S.E., Colombo J., Gajewski B.J., Gustafson K.M., Mundy D., Yeast J., Georgieff M.K., Markley L.A., Kerling E.H., Shaddy D.J. (2013). DHA Supplementation and Pregnancy Outcomes. Am. J. Clin. Nutr..

[232] Colombo J., Shaddy D.J., Gustafson K., Gajewski B.J., Thodosoff J.M., Kerling E., Carlson S.E. (2019). The Kansas University DHA Outcomes Study (KUDOS) Clinical Trial: Long-Term Behavioral Follow-up of the Effects of Prenatal DHA Supplementation. Am. J. Clin. Nutr..

[233] Scholtz S.A., Kerling E.H., Shaddy D.J., Li S., Thodosoff J.M., Colombo J., Carlson S.E. (2015). Docosahexaenoic Acid (DHA) Supplementation in Pregnancy Differentially Modulates Arachidonic Acid and DHA Status across FADS Genotypes in Pregnancy. Prostaglandins Leukot Essent Fat. Acids.

[234] Richardson A.J., Burton J.R., Sewell R.P., Spreckelsen T.F., Montgomery P. (2012). Docosahexaenoic Acid for Reading, Cognition and Behavior in Children Aged 7–9 Years: A Randomized, Controlled Trial (the DOLAB Study). PLoS ONE.

[235] Montgomery P., Burton J.R., Sewell R.P., Spreckelsen T.F., Richardson A.J. (2014). Fatty Acids and Sleep in UK Children: Subjective and Pilot Objective Sleep Results from the DOLAB Stud—A Randomized Controlled Trial. J. Sleep Res..

[236] Montgomery P., Spreckelsen T.F., Burton A., Burton J.R., Richardson A.J. (2018). Docosahexaenoic Acid for Reading, Working Memory and Behavior in UK Children Aged 7-9: A Randomized Controlled Trial for Replication (the DOLAB II Study). PLoS ONE.

[237] Muthayya S., Dwarkanath P., Thomas T., Ramprakash S., Mehra R., Mhaskar A., Mhaskar R., Thomas A., Bhat S., Vaz M. (2009). The Effect of Fish and ω-3 LCPUFA Intake on Low Birth Weight in Indian Pregnant Women. Eur. J. Clin. Nutr..

[238] Khandelwal S., Kondal D., Chaudhry M., Patil K., Swamy M.K., Pujeri G., Mane S.B., Kudachi Y., Gupta R., Ramakrishnan U. (2021). Prenatal Maternal Docosahexaenoic Acid (DHA) Supplementation and Newborn Anthropometry in India: Findings from DHANI. Nutrients.

[239] Bernstein A.M., Ding E.L., Willett W.C., Rimm E.B. (2012). A Meta-Analysis Shows That Docosahexaenoic Acid from Algal Oil Reduces Serum Triglycerides and Increases HDL-Cholesterol and LDL-Cholesterol in Persons without Coronary Heart Disease. J. Nutr..

[240] Rajaram S., Yip E.L., Reghunathan R., Mohan S., Sabaté J. (2017). Effect of Altering Dietary N-6:N-3 Polyunsaturated Fatty Acid Ratio with Plant and Marine-Based Supplement on Biomarkers of Bone Turnover in Healthy Adults. Nutrients.

[241] Dawczynski C., Dittrich M., Neumann T., Goetze K., Welzel A., Oelzner P., Völker S., Schaible A.M., Troisi F., Thomas L. (2018). Docosahexaenoic Acid in the Treatment of Rheumatoid Arthritis: A Double-Blind, Placebo-Controlled, Randomized Cross-over Study with Microalgae vs. Sunflower Oil. Clin. Nutr..

[242] López-Neyra A., Suárez L., Muñoz M., de Blas A., Ruiz de Valbuena M., Garriga M., Calvo J., Ribes C., Girón Moreno R., Máiz L. (2020). Long-Term Docosahexaenoic Acid (DHA) Supplementation in Cystic Fibrosis Patients: A Randomized, Multi-Center, Double-Blind, Placebo-Controlled Trial. Prostaglandins Leukot Essent Fat. Acids.

[243] Ochsenreither K., Glück C., Stressler T., Fischer L., Syldatk C. (2016). Production Strategies and Applications of Microbial Single Cell Oils. Front. Microbiol..

[244] Gema H., Kavadia A., Dimou D., Tsagou V., Komaitis M., Aggelis G. (2002). Production of γ-Linolenic Acid by Cunninghamella Echinulata Cultivated on Glucose and Orange Peel. Appl. Microbiol. Biotechnol..

[245] Xie D., Jackson E.N., Zhu Q. (2015). Sustainable Source of Omega-3 Eicosapentaenoic Acid from Metabolically Engineered Yarrowia Lipolytica: From Fundamental Research to Commercial Production. Appl. Microbiol. Biotechnol..

[246] Gemperlein K., Dietrich D., Kohlstedt M., Zipf G., Bernauer H.S., Wittmann C., Wenzel S.C., Müller R. (2019). Polyunsaturated Fatty Acid Production by Yarrowia Lipolytica Employing Designed Myxobacterial PUFA Synthases. Nat. Commun..

[247] Ledesma-Amaro R., Nicaud J.-M. (2016). Yarrowia Lipolytica as a Biotechnological Chassis to Produce Usual and Unusual Fatty Acids. Prog. Lipid Res..

[248] Cerone M., Smith T.K. (2021). A Brief Journey into the History of and Future Sources and Uses of Fatty Acids. Front. Nutr..

[249] Yu T., Zhou Y.J., Wenning L., Liu Q., Krivoruchko A., Siewers V., Nielsen J., David F. (2017). Metabolic Engineering of Saccharomyces Cerevisiae for Production of Very Long Chain Fatty Acid-Derived Chemicals. Nat. Commun..

[250] Ji X.-J., Huang H. (2019). Engineering Microbes to Produce Polyunsaturated Fatty Acids. Trends Biotechnol..

[251] Ratledge C., McNeil B., Archer D., Giavasis I., Harvey L. (2013). 19—Microbial Production of Polyunsaturated Fatty Acids as Nutraceuticals. Microbial Production of Food Ingredients, Enzymes and Nutraceuticals.

[252] Couto S.R., Sanromán M.Á. (2006). Application of Solid-State Fermentation to Food Industry—A Review. J. Food Eng..

[253] Shahidi F., CRC (2006). Nutraceutical and Specialty Lipids and Their Co-Products.

[254] Schwingshackl L., Hoffmann G. (2014). Monounsaturated Fatty Acids, Olive Oil and Health Status: A Systematic Review and Meta-Analysis of Cohort Studies. Lipids Health Dis..

[255] Beyzi E., Gunes A., Buyukkilic Beyzi S., Konca Y. (2019). Changes in Fatty Acid and Mineral Composition of Rapeseed (*Brassica napus ssp. oleifera* L.) Oil with Seed Sizes. Ind. Crops Prod..

[256] Kawakami Y., Yamanaka-Okumura H., Naniwa-Kuroki Y., Sakuma M., Taketani Y., Takeda E. (2015). Flaxseed Oil Intake Reduces Serum Small Dense Low-Density Lipoprotein Concentrations in Japanese Men: A Randomized, Double Blind, Crossover Study. Nutr. J..

[257] Hodson L., Crowe F.L., McLachlan K.J., Skeaff C.M. (2018). Effect of Supplementation with Flaxseed Oil and Different Doses of Fish Oil for 2 Weeks on Plasma Phosphatidylcholine Fatty Acids in Young Women. Eur. J. Clin. Nutr..

[258] Joris P.J., Draijer R., Fuchs D., Mensink R.P. (2020). Effect of α-Linolenic Acid on Vascular Function and Metabolic Risk Markers during the Fasting and Postprandial Phase: A Randomized Placebo-Controlled Trial in Untreated (Pre-)Hypertensive Individuals. Clin. Nutr..

[259] Kontogianni M.D., Vlassopoulos A., Gatzieva A., Farmaki A.-E., Katsiougiannis S., Panagiotakos D.B., Kalogeropoulos N., Skopouli F.N. (2013). Flaxseed Oil Does Not Affect Inflammatory Markers and Lipid Profile Compared to Olive Oil, in Young, Healthy, Normal Weight Adults. Metabolism.

[260] Raygan F., Taghizadeh M., Mirhosseini N., Akbari E., Bahmani F., Memarzadeh M.R., Sharifi N., Jafarnejad S., Banikazemi Z., Asemi Z. (2019). A Comparison between the Effects of Flaxseed Oil and Fish Oil Supplementation on Cardiovascular Health in Type 2 Diabetic Patients with Coronary Heart Disease: A Randomized, Double-Blinded, Placebo-Controlled Trial. Phytother. Res..

[261] Marineli R.D.S., Moraes É.A., Lenquiste S.A., Godoy A.T., Eberlin M.N., Maróstica M.R. (2014). Chemical Characterization and Antioxidant Potential of Chilean Chia Seeds and Oil (*Salvia Hispanica* L.). LWT—Food Sci. Technol..

[262] Knez Hrnčič M., Ivanovski M., Cör D., Knez Ž. (2020). Chia Seeds (*Salvia Hispanica* L.): An Overview—Phytochemical Profile, Isolation Methods, and Application. Molecules.

[263] EFSA Opinion on the Safety of ‘Chia Seeds (*Salvia hispanica* L.) and Ground Whole Chia Seeds’ as a Food Ingredient [1]. https://www.efsa.europa.eu/en/efsajournal/pub/996.

[264] Valenzuela R., Bascuñán K.A., Chamorro R., Barrera C., Sandoval J., Puigrredon C., Parraguez G., Orellana P., Gonzalez V., Valenzuela A. (2015). Modification of Docosahexaenoic Acid Composition of Milk from Nursing Women Who Received Alpha Linolenic Acid from Chia Oil during Gestation and Nursing. Nutrients.

[265] Vollmann J., Eynck C. (2015). Camelina as a Sustainable Oilseed Crop: Contributions of Plant Breeding and Genetic Engineering. Biotechnol. J..

[266] Manninen S., Lankinen M., de Mello V., Ågren J., Laaksonen D., Schwab U., Erkkilä A. (2019). The Effect of Camelina Sativa Oil and Fish Intakes on Fatty Acid Compositions of Blood Lipid Fractions. Nutr. Metab. Cardiovasc. Dis..

[267] Schwab U.S., Lankinen M.A., de Mello V.D., Manninen S.M., Kurl S., Pulkki K.J., Laaksonen D.E., Erkkilä A.T. (2018). Camelina Sativa Oil, but Not Fatty Fish or Lean Fish, Improves Serum Lipid Profile in Subjects with Impaired Glucose Metabolism-A Randomized Controlled Trial. Mol. Nutr. Food Res..

[268] Gonzales G.F., Gonzales C., Villegas L. (2014). Exposure of Fatty Acids after a Single Oral Administration of Sacha Inchi (*Plukenetia volubilis* L.) and Sunflower Oil in Human Adult Subjects. Toxicol. Mech. Methods.

[269] Nazir S., Wani I.A. (2021). Physicochemical Characterization of Basil (*Ocimum basilicum* L.) Seeds. J. Appl. Res. Med. Aromat. Plants.

[270] Kim D.-E., Shang X., Assefa A.D., Keum Y.-S., Saini R.K. (2018). Metabolite Profiling of Green, Green/Red, and Red Lettuce Cultivars: Variation in Health Beneficial Compounds and Antioxidant Potential. Food Res. Int..

[271] Nemzer B., Al-Taher F., Abshiru N. (2020). Phytochemical Composition and Nutritional Value of Different Plant Parts in Two Cultivated and Wild Purslane (*Portulaca oleracea* L.) Genotypes. Food Chem..

[272] Petropoulos S.A., Karkanis A., Fernandes Â., Barros L., Ferreira I.C.F.R., Ntatsi G., Petrotos K., Lykas C., Khah E. (2015). Chemical Composition and Yield of Six Genotypes of Common Purslane (*Portulaca oleracea* L.): An Alternative Source of Omega-3 Fatty Acids. Plant Foods Hum. Nutr..

[273] Burdge G.C., Calder P.C. (2005). Conversion of Alpha-Linolenic Acid to Longer-Chain Polyunsaturated Fatty Acids in Human Adults. Reprod Nutr. Dev..

[274] Prasad P., Sreedhar R.V. (2020). Identification and Functional Characterization of Buglossoides Arvensis Microsomal Fatty Acid Desaturation Pathway Genes Involved in Polyunsaturated Fatty Acid Synthesis in Seeds. J. Biotechnol..

[275] Harris W.S. (2012). Stearidonic Acid as a ‘pro-Eicosapentaenoic Acid’. Curr. Opin. Lipidol..

[276] Kuhnt K., Degen C., Jaudszus A., Jahreis G. (2012). Searching for Health Beneficial N-3 and n-6 Fatty Acids in Plant Seeds. Eur. J. Lipid Sci. Technol..

[277] Kriese U., Schumann E., Weber W.E., Beyer M., Brühl L. (2004). Matthäus Oil Content, Tocopherol Composition and Fatty Acid Patterns of the Seeds of 51 *Cannabis Sativa* L. Genotypes. Euphytica.

[278] Oomah B.D., Busson M., Godfrey D.V., Drover J.C.G. (2002). Characteristics of Hemp (*Cannabis sativa* L.) Seed Oil. Food Chem..

[279] Prasad P., Anjali P., Sreedhar R.V. (2021). Plant-Based Stearidonic Acid as Sustainable Source of Omega-3 Fatty Acid with Functional Outcomes on Human Health. Crit. Rev. Food Sci. Nutr..

[280] James M.J., Ursin V.M., Cleland L.G. (2003). Metabolism of Stearidonic Acid in Human Subjects: Comparison with the Metabolism of Other N−3 Fatty Acids. Am. J. Clin. Nutr..

[281] Kuhnt K., Weiß S., Kiehntopf M., Jahreis G. (2016). Consumption of Echium Oil Increases EPA and DPA in Blood Fractions More Efficiently Compared to Linseed Oil in Humans. Lipids Health Dis..

[282] Lemke S.L., Vicini J.L., Su H., Goldstein D.A., Nemeth M.A., Krul E.S., Harris W.S. (2010). Dietary Intake of Stearidonic Acid–Enriched Soybean Oil Increases the Omega-3 Index: Randomized, Double-Blind Clinical Study of Efficacy and Safety. Am. J. Clin. Nutr..

[283] Surette M.E., Edens M., Chilton F.H., Tramposch K.M. (2004). Dietary Echium Oil Increases Plasma and Neutrophil Long-Chain (n-3) Fatty Acids and Lowers Serum Triacylglycerols in Hypertriglyceridemic Humans. J. Nutr..

[284] Harris W.S., Lemke S.L., Hansen S.N., Goldstein D.A., DiRienzo M.A., Su H., Nemeth M.A., Taylor M.L., Ahmed G., George C. (2008). Stearidonic Acid-Enriched Soybean Oil Increased the Omega-3 Index, an Emerging Cardiovascular Risk Marker. Lipids.

[285] Krul E.S., Lemke S.L., Mukherjea R., Taylor M.L., Goldstein D.A., Su H., Liu P., Lawless A., Harris W.S., Maki K.C. (2012). Effects of Duration of Treatment and Dosage of Eicosapentaenoic Acid and Stearidonic Acid on Red Blood Cell Eicosapentaenoic Acid Content. Prostaglandins Leukot Essent Fat. Acids.

[286] Goyens P.L.L., Spilker M.E., Zock P.L., Katan M.B., Mensink R.P. (2006). Conversion of Alpha-Linolenic Acid in Humans Is Influenced by the Absolute Amounts of Alpha-Linolenic Acid and Linoleic Acid in the Diet and Not by Their Ratio. Am. J. Clin. Nutr..

[287] Guil-Guerrero J.L., Gómez-Mercado F., Ramos-Bueno R.P., Rincón-Cervera M.Á., Venegas-Venegas E. (2014). Restricted-Range Boraginaceae Species Constitute Potential Sources of Valuable Fatty Acids. J. Am. Oil Chem. Soc..

[288] EFSA Scientific Opinion on the Substantiation of Health Claims Related to Echium Oil and Maintenance of Normal Blood Concentrations of Triglycerides (ID 548) Pursuant to Article 13(1) of Regulation (EC) No 1924/2006. https://www.efsa.europa.eu/en/efsajournal/pub/1256.

[289] Cumberford G., Hebard A. (2015). Ahiflower Oil: A Novel Non-GM Plant-Based Omega-3 + 6 Source. Lipid Technol..

[290] Sreedhar R.V., Prasad P., Reddy L.P.A., Rajasekharan R., Srinivasan M. (2017). Unravelling a Stearidonic Acid-Rich Triacylglycerol Biosynthetic Pathway in the Developing Seeds of Buglossoides Arvensis: A Transcriptomic Landscape. Sci. Rep..

[291] EFSA Scientific Opinion on the Safety of Refined Buglossoides Oil as a Novel Food Ingredient. https://www.efsa.europa.eu/it/efsajournal/pub/4029.

[292] GRAS Notices GRN No. 486 Oil from the Seeds of Buglossoides Arvensis. https://www.cfsanappsexternal.fda.gov/scripts/fdcc/index.cfm?set=GRASNotices&id=486.

[293] Lefort N., LeBlanc R., Giroux M.-A., Surette M.E. (2016). Consumption of Buglossoides Arvensis Seed Oil Is Safe and Increases Tissue Long-Chain n-3 Fatty Acid Content More than Flax Seed Oil—Results of a Phase I Randomised Clinical Trial. J. Nutr. Sci..

[294] Lefort N., LeBlanc R., Surette M.E. (2017). Dietary Buglossoides Arvensis Oil Increases Circulating N-3 Polyunsaturated Fatty Acids in a Dose-Dependent Manner and Enhances Lipopolysaccharide-Stimulated Whole Blood Interleukin-10—A Randomized Placebo-Controlled Trial. Nutrients.

[295] Guil-Guerrero J.L., González-Fernández M.J., Lyashenko S., Fabrikov D., Rincón-Cervera M.Á., Urrestarazu M., Gómez-Mercado F. (2020). γ-Linolenic and Stearidonic Acids from Boraginaceae of Diverse Mediterranean Origin. Chem. Biodivers.

[296] Piskernik S., Vidrih R., Demšar L., Koron D., Rogelj M., Žontar T.P. (2018). Fatty Acid Profiles of Seeds from Different Ribes Species. LWT.

[297] Schulze M.B., Minihane A.M., Saleh R.N.M., Risérus U. (2020). Intake and Metabolism of Omega-3 and Omega-6 Polyunsaturated Fatty Acids: Nutritional Implications for Cardiometabolic Diseases. Lancet Diabetes Endocrinol..

[298] Hall M.N., Campos H., Li H., Sesso H.D., Stampfer M.J., Willett W.C., Ma J. (2007). Blood Levels of Long-Chain Polyunsaturated Fatty Acids, Aspirin, and the Risk of Colorectal Cancer. Cancer Epidemiol. Biomark. Prev..

[299] Walker C.G., Jebb S.A., Calder P.C. (2013). Stearidonic Acid as a Supplemental Source of ω-3 Polyunsaturated Fatty Acids to Enhance Status for Improved Human Health. Nutrition.

[300] Calder P.C. (2006). N−3 Polyunsaturated Fatty Acids, Inflammation, and Inflammatory Diseases. Am. J. Clin. Nutr..

[301] Whelan J. (2009). Dietary Stearidonic Acid Is a Long Chain (n-3) Polyunsaturated Fatty Acid with Potential Health Benefits. J. Nutr..

[302] Ohnishi H., Saito Y. (2013). Eicosapentaenoic Acid (EPA) Reduces Cardiovascular Events: Relationship with the EPA/Arachidonic Acid Ratio. J. Atheroscler. Thromb..

[303] Greupner T., Koch E., Kutzner L., Hahn A., Schebb N.H., Schuchardt J.P. (2019). Single-Dose SDA-Rich Echium Oil Increases Plasma EPA, DPAn3, and DHA Concentrations. Nutrients.

[304] Miles E.A., Banerjee T., Calder P.C. (2004). The Influence of Different Combinations of Gamma-Linolenic, Stearidonic and Eicosapentaenoic Acids on the Fatty Acid Composition of Blood Lipids and Mononuclear Cells in Human Volunteers. Prostaglandins Leukot Essent Fat. Acids.

[305] Arterburn L.M., Hall E.B., Oken H. (2006). Distribution, Interconversion, and Dose Response of n-3 Fatty Acids in Humans. Am. J. Clin. Nutr..

[306] Sands S.A., Reid K.J., Windsor S.L., Harris W.S. (2005). The Impact of Age, Body Mass Index, and Fish Intake on the EPA and DHA Content of Human Erythrocytes. Lipids.

[307] Pottel L., Lycke M., Boterberg T., Pottel H., Goethals L., Duprez F., Maes A., Goemaere S., Rottey S., Foubert I. (2014). Echium Oil Is Not Protective against Weight Loss in Head and Neck Cancer Patients Undergoing Curative Radio(Chemo)Therapy: A Randomised-Controlled Trial. BMC Complement Altern. Med..

[308] Pieters D.J.M., Mensink R.P. (2015). Effects of Stearidonic Acid on Serum Triacylglycerol Concentrations in Overweight and Obese Subjects: A Randomized Controlled Trial. Eur. J. Clin. Nutr..

[309] Ursin V.M. (2003). Modification of Plant Lipids for Human Health: Development of Functional Land-Based Omega-3 Fatty Acids. J. Nutr..

[310] Lee T.C., Ivester P., Hester A.G., Sergeant S., Case L.D., Morgan T., Kouba E.O., Chilton F.H. (2014). The Impact of Polyunsaturated Fatty Acid-Based Dietary Supplements on Disease Biomarkers in a Metabolic Syndrome/Diabetes Population. Lipids Health Dis..

[311] Diwakar B.T., Dutta P.K., Lokesh B.R., Naidu K.A. (2010). Physicochemical Properties of Garden Cress (*Lepidium Sativum* L.) Seed Oil. J. Am. Oil Chem. Soc..

[312] Amjad Khan W., Chun-Mei H., Khan N., Iqbal A., Lyu S.-W., Shah F. (2017). Bioengineered Plants Can Be a Useful Source of Omega-3 Fatty Acids. BioMed Res. Int..

[313] Ohlrogge J.B. (1994). Design of New Plant Products: Engineering of Fatty Acid Metabolism. Plant Physiol..

[314] Napier J.A., Usher S., Haslam R.P., Ruiz-Lopez N., Sayanova O. (2015). Transgenic Plants as a Sustainable, Terrestrial Source of Fish Oils. Eur. J. Lipid Sci. Technol..

[315] Ruiz-López N., Haslam R.P., Venegas-Calerón M., Li T., Bauer J., Napier J.A., Sayanova O. (2012). Enhancing the Accumulation of Omega-3 Long Chain Polyunsaturated Fatty Acids in Transgenic Arabidopsis Thaliana via Iterative Metabolic Engineering and Genetic Crossing. Transgenic. Res..

[316] Kajikawa M., Matsui K., Ochiai M., Tanaka Y., Kita Y., Ishimoto M., Kohzu Y., Shoji S., Yamato K.T., Ohoyama K. (2008). Production of Arachidonic and Eicosapentaenoic Acids in Plants Using Bryophyte Fatty Acid Δ6-Desaturase, Δ6-Elongase, and Δ5-Desaturase Genes. Biosci. Biotechnol. Biochem..

[317] Enzing C., Ploeg M., Barbosa M., Sijtsma L. Microalgae-Based Products for the Food and Feed Sector: An Outlook for Europe. https://publications.jrc.ec.europa.eu/repository/handle/JRC85709.

[318] Feednavigator.com ‘Alltech Has Not Exited Algae’. https://www.feednavigator.com/Article/2018/08/29/Alltech-has-not-exited-algae.

[319] Napier J.A., Olsen R.-E., Tocher D.R. (2019). Update on GM Canola Crops as Novel Sources of Omega-3 Fish Oils. Plant Biotechnol. J..

[320] Mansour M.P., Shrestha P., Belide S., Petrie J.R., Nichols P.D., Singh S.P. (2014). Characterization of Oilseed Lipids from “DHA-Producing Camelina Sativa”: A New Transformed Land Plant Containing Long-Chain Omega-3 Oils. Nutrients.

[321] Petrie J.R., Shrestha P., Belide S., Kennedy Y., Lester G., Liu Q., Divi U.K., Mulder R.J., Mansour M.P., Nichols P.D. (2014). Metabolic Engineering Camelina Sativa with Fish Oil-Like Levels of DHA. PLoS ONE.

[322] Ruiz-Lopez N., Haslam R.P., Napier J.A., Sayanova O. (2014). Successful High-Level Accumulation of Fish Oil Omega-3 Long-Chain Polyunsaturated Fatty Acids in a Transgenic Oilseed Crop. Plant J..

[323] Petrie J.R., Shrestha P., Zhou X.-R., Mansour M.P., Liu Q., Belide S., Nichols P.D., Singh S.P. (2012). Metabolic Engineering Plant Seeds with Fish Oil-like Levels of DHA. PLoS ONE.

[324] West A.L., Miles E.A., Lillycrop K.A., Han L., Sayanova O., Napier J.A., Calder P.C., Burdge G.C. (2019). Postprandial Incorporation of EPA and DHA from Transgenic Camelina Sativa Oil into Blood Lipids Is Equivalent to That from Fish Oil in Healthy Humans. Br. J. Nutr..

[325] West A.L., Miles E.A., Lillycrop K.A., Han L., Napier J.A., Calder P.C., Burdge G.C. (2020). Dietary Supplementation with Seed Oil from Transgenic Camelina Sativa Induces Similar Increments in Plasma and Erythrocyte DHA and EPA to Fish Oil in Healthy Humans. Br. J. Nutr..

[326] Ruiz-López N., Sayanova O., Napier J.A., Haslam R.P. (2012). Metabolic Engineering of the Omega-3 Long Chain Polyunsaturated Fatty Acid Biosynthetic Pathway into Transgenic Plants. J. Exp. Bot..

[327] Desaint N., Varbanova M. (2013). The Use and Value of Polling to Determine Public Opinion on GMOs in Europe. GM Crops Food.

[328] Lucht J.M. (2015). Public Acceptance of Plant Biotechnology and GM Crops. Viruses.

[329] Ruiz-López N., Haslam R.P., Venegas-Calerón M., Larson T.R., Graham I.A., Napier J.A., Sayanova O. (2009). The Synthesis and Accumulation of Stearidonic Acid in Transgenic Plants: A Novel Source of ‘Heart-Healthy’ Omega-3 Fatty Acids. Plant Biotechnol. J..

[330] Eckert H., LaVallee B., Schweiger B.J., Kinney A.J., Cahoon E.B., Clemente T. (2006). Co-Expression of the Borage Δ6 Desaturase and the Arabidopsis Δ15 Desaturase Results in High Accumulation of Stearidonic Acid in the Seeds of Transgenic Soybean. Planta.

[331] Harris W.S. (2012). Stearidonic Acid-Enhanced Soybean Oil: A Plant-Based Source of (n-3) Fatty Acids for Foods. J. Nutr..

[332] Lee K.R., Kim K.H., Kim J.B., Hong S.B., Jeon I., Kim H.U., Lee M.H., Kim J.K. (2019). High Accumulation of γ-Linolenic Acid and Stearidonic Acid in Transgenic Perilla (Perilla Frutescens Var. Frutescens) Seeds. BMC Plant Biol..

[333] Lemke S.L., Maki K.C., Hughes G., Taylor M.L., Krul E.S., Goldstein D.A., Su H., Rains T.M., Mukherjea R. (2013). Consumption of Stearidonic Acid-Rich Oil in Foods Increases Red Blood Cell Eicosapentaenoic Acid. J. Acad. Nutr. Diet..

[334] GRAS Notices GRN No. 283 Stearidonic Acid Soybean Oil. https://www.cfsanappsexternal.fda.gov/scripts/fdcc/?set=GRASNotices&id=283&sort=GRN_No&order=DESC&startrow=1&type=basic&search=soybean.

[335] EFSA Statement Complementing the EFSA Scientific Opinion on Application (EFSA-GMO-NL-2010-85) for Authorisation of Food and Feed Containing, Consisting of and Produced from Genetically Modified Soybean MON 87769 × MON 89788. https://www.efsa.europa.eu/it/efsajournal/pub/6589.

[336] EFSA Petition for the Determination of Nonregulated Status for EPA+DHA Canola Event LBFLFK. https://www.regulations.gov/document/APHIS-2018-0014-0002.

[337] Eck C. BASF Petition (17-321-01p) for Determination of Nonregulated Status of EPA + DHA Canola Event LBFLFK. https://www.aphis.usda.gov/brs/aphisdocs/17_32101p_fpra.pdf.

[338] Abbadi A., Domergue F., Bauer J., Napier J.A., Welti R., Zähringer U., Cirpus P., Heinz E. (2004). Biosynthesis of Very-Long-Chain Polyunsaturated Fatty Acids in Transgenic Oilseeds: Constraints on Their Accumulation. Plant Cell.

[339] Cheng B., Wu G., Vrinten P., Falk K., Bauer J., Qiu X. (2010). Towards the Production of High Levels of Eicosapentaenoic Acid in Transgenic Plants: The Effects of Different Host Species, Genes and Promoters. Transgenic. Res..

[340] Robert S.S., Singh S.P., Zhou X.R., Petrie J.R., Blackburn S.I., Mansour P.M., Nichols P.D., Liu Q., Green A.G. (2005). Metabolic Engineering of Arabidopsis to Produce Nutritionally Important DHA in Seed Oil. Funct. Plant Biol..

